# Triglyceride-glucose index, symptomatic intracranial artery stenosis and recurrence risk in minor stroke patients with hypertension

**DOI:** 10.1186/s12933-023-01823-6

**Published:** 2023-04-19

**Authors:** Yongle Wang, Tingting Liu, Yanan Li, Kaili Zhang, Haimei Fan, Jing Ren, Juan Li, Yali Li, Xinyi Li, Xuemei Wu, Junhui Wang, Lixi Xue, Xiaolei Gao, Yuping Yan, Gaimei Li, Qingping Liu, Wenhua Niu, Wenxian Du, Yuting Liu, Xiaoyuan Niu

**Affiliations:** 1 Department of Neurology, First Hospital of Shanxi Medical University, No. 85, Jiefangnan Street, Yingze District, Taiyuan, Shanxi China; 2 Clinical College, Shanxi Medical University, No. 58, Xinjiannan Street, Yingze District, Taiyuan, Shanxi China; 3 Department of Neurology, Shanxi Bethune Hospital, Tongji Shanxi Hospital, Shanxi Academy of Medical Sciences, Third Hospital of Shanxi Medical University, Taiyuan, Shanxi China; 4 Department of Neurology, Sixth Hospital of Shanxi Medical University (General Hospital of Tisco), Taiyuan, Shanxi China; 5 Shanxi Province Cardiovascular Disease Hospital, Taiyuan, Shanxi China; 6 Cangzhou Central Hospital, Cangzhou, Hebei China; 7 Yanhu Branch First Hospital of Shanxi Medical University, Yuncheng, Shanxi China; 8 Taiyuan Wanbailin District Medical Group Central Hospital, Taiyuan, Shanxi China; 9 China Railway 17th Bureau Group Company Central Hospital, Shanxi Taiyuan, China; 10 First People’s Hospital of JIN ZHONG, Jinzhong, Shanxi China

**Keywords:** Triglyceride-glucose index, Symptomatic intracranial atherosclerosis, Stroke recurrence, Insulin resistance

## Abstract

**Background:**

The triglyceride-glucose (TyG) index, a simple measure of insulin resistance, is associated with intracranial atherosclerosis (ICAS) and stroke. In hypertensive populations, this association may be pronounced. The aim was to investigate the relationship between TyG and symptomatic intracranial atherosclerosis (sICAS) and recurrence risk in ischemic stroke patients with hypertension.

**Methods:**

This prospective, multicenter cohort study included patients with acute minor ischemic stroke with a preadmission diagnosis of hypertension from September 2019 to November 2021 with a 3-month follow-up. The presence of sICAS was determined by a combination of clinical manifestations, the location of the infarction, and the corresponding artery with moderate-to-severe stenosis. ICAS burden was determined by the degree and number of ICAS occurrences. Fasting blood glucose (FBG) and triglyceride (TG) were measured to calculate TyG. The main outcome was ischemic stroke recurrence during the 90-day follow-up. Multivariate regression models were used to explore the association of TyG, sICAS, and ICAS burden with stroke recurrence.

**Results:**

There were 1281 patients with a mean age of 61.6 ± 11.6 years; 70.1% were male, and 26.4% were diagnosed with sICAS. There were 117 patients who experienced stroke recurrence during follow-up. Patients were categorized according to quartiles of TyG. After adjusting for confounders, the risk of sICAS was greater (OR 1.59, 95% CI 1.04–2.43, p = 0.033) and the risk of stroke recurrence was significantly higher (HR 2.02, 95% CI 1.07–3.84, p = 0.025) in the fourth TyG quartile than in the first quartile. The restricted cubic spline (RCS) plot revealed a linear relationship between TyG and sICAS, and the threshold value for TyG was 8.4. Patients were then dichotomized into low and high TyG groups by the threshold. Patients with high TyG combined with sICAS had a higher risk of recurrence (HR 2.54, 95% CI 1.39–4.65) than patients with low TyG without sICAS. An interaction effect on stroke recurrence between TyG and sICAS was found (p = 0.043).

**Conclusion:**

TyG is a significant risk factor for sICAS in hypertensive patients, and there is a synergistic effect of sICAS and higher TyG on ischemic stroke recurrence.

*Trial registration number:* The study was registered on 16 August 2019 at https://www.chictr.org.cn/showprojen.aspx?proj=41160 (No. ChiCTR1900025214).

**Supplementary Information:**

The online version contains supplementary material available at 10.1186/s12933-023-01823-6.

## Background

Intracranial atherosclerosis (ICAS) is one of the major causes of ischemic stroke, particularly among Eastern Asian populations [1, 2]. Individuals with symptomatic intracranial artery stenosis (sICAS) are at high risk for recurrent stroke in the short term [3]. Previous studies have identified insulin resistance (IR) and hypertension as significant risk factors for the development of sICAS [4, 5]. In the context of endothelial dysfunction, vascular remodeling, and arterial stiffness associated with hypertension, IR-induced lipid metabolism disorders accelerate atherosclerosis formation [6, 7]. Hypertension and insulin resistance can synergistically promote atherosclerosis progression, increasing the risk of ischemic disease [8]. Consequently, managing insulin resistance is a critical concern for preventing atherosclerosis in hypertensive populations.

The TyG index, derived from triglyceride (TG) and fasting blood glucose (FBG) levels, has been established as a simple and reliable tool to measure IR [9]. Several studies have demonstrated the feasibility and validity of using TyG to predict the severity of atherosclerosis [10]. Furthermore, TyG is a more accurate predictor of cardiovascular disease than traditional vascular risk factors [11]. TyG has been shown to be significantly associated with the incidence and poor prognosis of stroke [12, 13].

Although TyG has been established as a predictor of atherosclerosis severity and cardiovascular disease, its ability to predict sICAS development in hypertensive populations is uncertain. It is also unclear whether the presence of both IR and sICAS has a synergistic effect on stroke recurrence. Thus, the objective of this study is to investigate whether higher TyG levels increase the risk of sICAS development in hypertensive patients and to determine whether the combined presence of sICAS and higher TyG levels increases the risk of short-term stroke recurrence.

## Methods

This research involves a secondary analysis of a multicenter prospective cohort entitled “Safety and efficacy of aspirin-clopidogrel in acute noncardiogenic minor ischemic stroke: a prospective and multicenter study based on real-world (SEACOAST)”. The SEACOAST study included a total of eight stroke centers in China and enrolled patients with a National Institutes of Health Stroke Scale (NIHSS) ≤ 5 within 72 h of stroke onset between September 2019 and November 2021.The study protocol is provided in the supplement. The study was registered at https://www.chictr.org.cn/showprojen.aspx?proj=41160 (No. ChiCTR1900025214). The Ethics Committee of the First Hospital of Shanxi Medical University approved the study, and all patients provided written informed consent before enrollment. The study was conducted in two parts: first, to analyze the correlation between TyG and sICAS, as well as the burden of ICAS; second, to further analyze the synergistic effect of sICAS and TyG on 90-day ischemic stroke recurrence.

### Inclusion and exclusion criteria

The study inclusion criteria were as follows: 1. acute ischemic stroke within 72 h from stroke onset (last seen as normal) to hospital arrival; 2. NIHSS <  = 5 at presentation; 3. intracranial vascular examination: magnetic resonance angiography (MRA), computed tomographic arteriography (CTA) or digital subtraction angiography (DSA) completed in the emergency room or during hospitalization; 4. laboratory tests, including FBG and TG, completed the next morning after admission; and 5. previously diagnosed hypertension told by doctor or taking antihypertensive medication two weeks before stroke onset.

The exclusion criteria were as follows: 1. modified Rankin score (mRs) > 2 before onset; 2. anticoagulation therapy (due to atrial fibrillation, venous thrombosis, etc.) at admission and follow-up period; 3. endovascular or surgical treatment during hospitalization and 3-month follow-up; 4. cardiogenic stroke; 5. transient ischemic attack (TIA); 6. baseline computed tomography (CT) or magnetic resonance imaging (MRI) findings of new cerebral hemorrhage, tumor, vascular malformation, and other nonischemic events; 7. emergency treatment with thrombolysis or endovascular thrombectomy; 8. participation in other clinical studies during the same period; and 9. severe mental disorders and other serious systemic diseases with a life expectancy of less than 3 months.

### Data collection

The study collected baseline information, past history, clinical features of stroke, laboratory findings, imaging features, and medication use during hospitalization from patients enrolled in the study. The data were collected prospectively at each center and recorded in a web-based registry database based on the Research Electronic Data Capture system [14]. Quality control was performed monthly, and trained clinicians were responsible for data entry. Variables for analysis in this study included age, sex, body mass index (BMI), systolic blood pressure (SBP) and diastolic blood pressure (DBP) at admission, prior mRS score, NIHSS score at presentation, time from onset to hospital arrival, smoking status (never/quit/currently smoking), drinking, and past medical history (hypertension, diabetes mellitus, dyslipidemia, atrial fibrillation, TIA, ischemic stroke, carotid atherosclerosis). Laboratory tests included TG, low-density lipoprotein cholesterol (LDL-C), high-density lipoprotein cholesterol (HDL-C), total cholesterol (TC), FBG, homocysteine (HCY), creatinine, urea, platelets (PLTs), white blood cells (WBCs), and uric acid. Treatment during hospitalization included antiplatelet therapy (single antiplatelet/dual antiplatelet drugs), statin therapy (no statin or received either atorvastatin 40–80 mg or rosuvastatin 20 mg), antihypertensive treatment (yes/no) and hypoglycemic treatment (yes/no).

Imaging features were obtained from cranial MRI, CT, CTA and DSA during emergency or hospitalization. Cranial MRI included T1-weighted imaging, T2-weighted imaging, diffusion-weighted imaging (DWI), and MRA sequences. CTA, MRA and DSA were used to evaluate the location and degree of ICAS and to determine the location of sICAS in conjunction with the clinical presentation and location of the infarct lesion on DWI. Intracranial arteries, including the intracranial segment of the internal carotid artery (ICA), the M1 and M2 segments of the middle cerebral artery (MCA), the anterior cerebral artery (ACA), the P1 and P2 segments of the posterior cerebral artery (PCA), the V4 segment of the vertebral artery (VA), and the basilar artery (BA), were evaluated for stenosis. The degree of stenosis was evaluated using the Warfarin Aspirin Symptomatic Intracranial Disease (WASID) criteria (between the narrowest point and compared with the normal luminal size before the stenosis), where stenosis less than 50% was considered mild, 50% to 69% was considered moderate, and 70 to 99% was considered severe. and 70–99% as severe stenosis [15]. The total burden of ICAS was assessed using the “intracranial atherosclerosis burden (ICASB) score”, proposed by Wang et al. [16]. The ICASB score is a rating method where the stenosis degree of each intracranial artery is measured, and then a value was assigned as follows: 0 for no stenosis; 1 for mild stenosis; 2 for moderate stenosis; and 3 for severe stenosis or occlusion. The cumulative score for all arteries is calculated as ICASB, which is further divided into three subgroups: Low (< 4 points), Medium (4–5 points), and High (> 5 points). Multiple infarctions were defined as at least two distinct lesions on DWI, separated in space or discrete on contiguous slices.

Blood samples were collected from the anterior elbow vein of all patients in the morning after an overnight fasting for at least 8 h. FBG and TG were measured in a biochemical laboratory in each study center. TyG was calculated as follows: ln (fasting TG [mg/dL] * FBG [mg/dL]/2) [17].

### Study outcome

The study was conducted in two parts. The outcomes for the first part were the presence of sICAS and burden of ICAS evaluated after admission. The presence of sICAS was defined as a stenosis of > 50% of an intracranial artery, accompanied by clinical symptoms and DWI lesions that matched the artery [18]. ICASB was used to evaluate the burden of ICAS and further details can be found in the data collection. In the second part, the outcome was ischemic stroke recurrence within 90 days of symptom onset. Ischemic stroke recurrence was defined as acute new focal neurologic deficit lasting for more than 24 h, with an increase in the NIHSS score of four or more, or imaging evidence on MRI or CT (new infarction or enlargement of the original infarction area) [19]. Follow-up information was obtained via telephone or querying the visit records during the follow-up period. Trained telephone interviewers used a standardized structured follow-up questionnaire to ensure the quality of telephone follow-up.

### Statistical methods

Categorical variables were presented as frequencies (percentages), and continuous variables were presented as mean (standard deviation) or median (interquartile range). The differences between groups for categorical variables were analyzed using Pearson’s chi-squared test, and for continuous variables between two groups, t-test or Wilcoxon rank sum test was used depending on the normal distribution of samples. Comparisons between three or more groups were made using ANOVA (parametric test) or the Kruskal‒Wallis test (nonparametric test). Logistic regression/linear regression models were used to analyze the relationship between TyG and sICAS/ICASB, with confounders selected based on univariate analysis and previous literature reports. The adjusted models included model I (adjusted for age and sex), and model II (further adjusted for BMI, SBP, DBP, smoking status, drinking, previous stroke, and previous diabetes mellitus). Contour plots [20] were constructed to visually reflect whether different baseline characteristics (age, BMI, SBP and DBP) modified the relationship between TyG and sICAS. Cox regression models were used to analyze the effects of TyG, sICAS, and ICASB on ischemic stroke recurrence, with model I (adjusted for age and sex), model II (further adjusted for BMI, SBP, smoking status, time at onset, NIHSS score at arrival, WBC count, previous stroke, and previous diabetes mellitus based on model I), and model III (further adjusted for treatment during admission including antiplatelet therapy, statin therapy, antihypertensive treatment and hypoglycemic treatment based on model II) being applied. The dose‒response relationship of TyG and ICASB to stroke recurrence was explored using restricted cubic spline (RCS) plots. A model with three knots located at the 5th, 50th, and 95th percentiles was applied, and a nonlinear relationship was examined. The optimal cutoff value of TyG that distinguished a higher risk of stroke recurrence was determined using X-tile software [21]. Based on this cutoff point, the patients were then divided into two groups: a low TyG group and a high TyG group. Kaplan‒Meier curves were plotted to present the survival status of patients with or without sICAS at high or low TyG levels and were examined by the log-rank test. To further determine the synergistic relationship between TyG and sICAS, the interaction term between TyG and the presence of sICAS on stroke recurrence was calculated. Integrated discrimination improvement (IDI) and category-free net reclassification improvement (NRI) were applied to investigate the incremental prognostic effect of the TyG index on stroke recurrence beyond traditional risk factors. The data were missing at random (Additional file 1: Fig. S1), and missing data were processed with multiple imputation with chained equations, and 5 replications were produced to process the missing data on WBC, BMI, PLT, smoking status, drinking, HCY, HDL, SBP, DBP, LDL and TC (Additional file 1: Table S1). A sensitivity analysis was performed after the imputation to examine the robustness of the main analysis. All tests were two-sided, and a P value of 0.05 was considered a significant difference. All analyses were performed by R 4.2.1 (http://www.R-project.org, The R Foundation).

## Results

### Baseline information

**Fig. 1:**
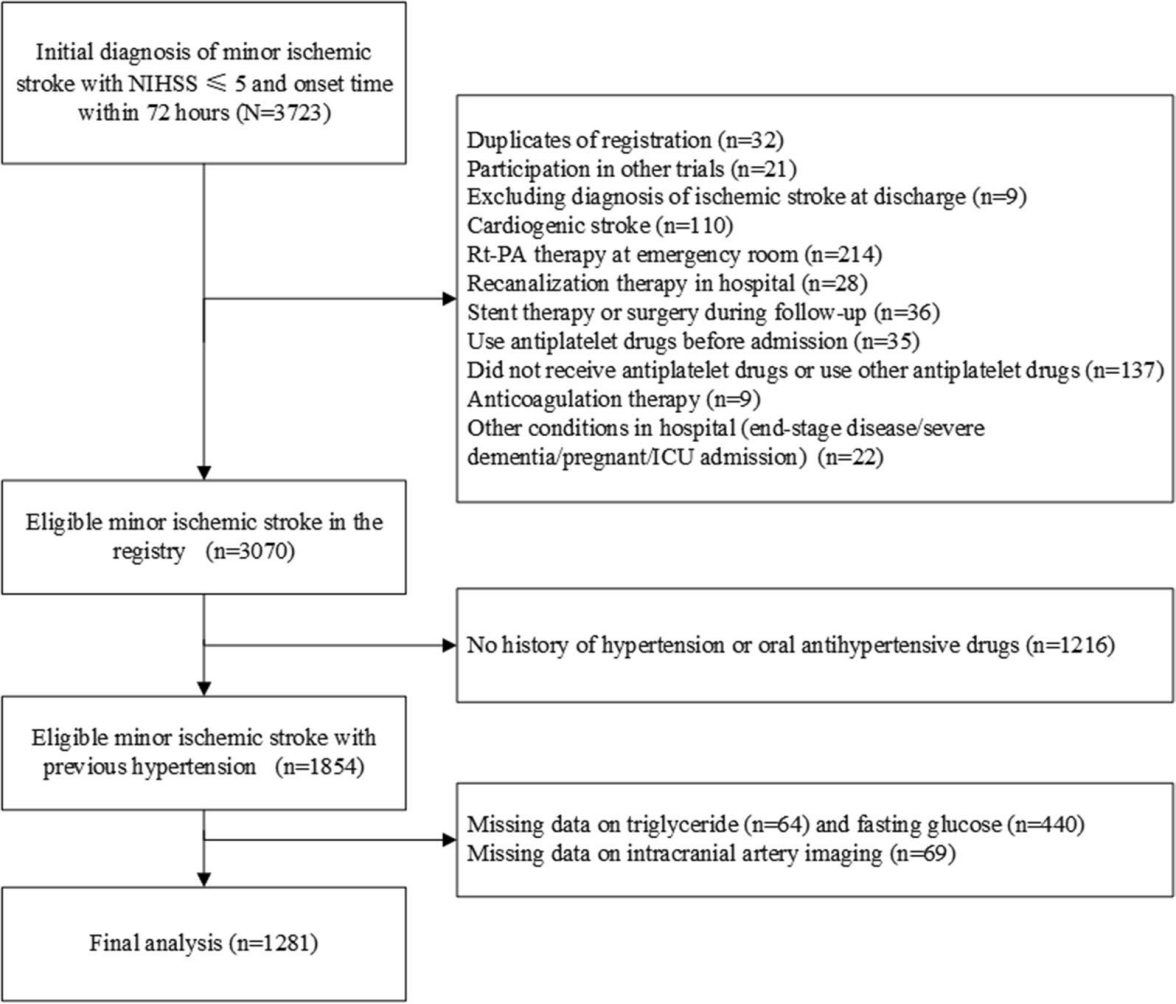
Study flowchart. A total of 1281 patients were included in the final analysis

A total of 1281 patients were included in the study. The detailed inclusion flow chart is shown in Fig. 1. Patients without records of TG, FBG and intracranial artery imaging in the registry were excluded from the analysis. The baseline characteristics of patients included and excluded are shown in Additional file 1: Table S2, which were balanced except for a lower age, higher BMI, lower mRs, more current smokers, more probability of drinking, less chance of intensive statin therapy, and higher LDL, TC, HCY, WBC and neutrophil levels in the included population. These variables could be considered potential confounders in the next regression analyses. In the included population, the mean age of patients was 61.6 years, and 70.1% of the population was male. The median NIHSS score was 2. Patients were admitted to the hospital on average at 25.7 h after onset. Patients in the 4th quantile of TyG were younger, had higher BMI, higher blood pressure on admission, higher rates of previously diagnosed diabetes and dyslipidemia, and lower rates of previous ischemic stroke and carotid stenosis. Regarding laboratory tests, patients in the 4th quantile of TyG had higher TG, LDL-C, TC, FBG, PLT, and WBC, while HDL-C and HCY were relatively lower. In-hospital treatment was well balanced, except for a higher rate of hypoglycemic therapy in the 4th TyG quantile. In terms of imaging features, patients in the 4th TyG quantile had higher ICASB scores and a higher probability of sICAS, while the proportion of multiple infarctions on DWI was balanced among groups (Table 1). The baseline information of patients with and without sICAS was presented in Additional file 1: Table S3. Patients with sICAS were older, had a lower proportion of men, higher NIHSS scores, LDL-C levels, WBC, neutrophil levels, TyG levels, higher risk of multiple infarcts and higher ICASB scores. A higher proportion of sICAS patients were treated with intensive statin therapy after hospitalization.

**Table 1:** Baseline characteristics of quartiles of TyG

	Total (n = 1281)	TyG^#^ quartile 1 (n = 320)	TyG quartile 2 (n = 320)	TyG quartile 3 (n = 320)	TyG quartile 4 (n = 321)	P^*^ value	P^**^ value
Age, mean (SD), year	61.6 (11.6)	64.6 (11.3)	62.9 (12.3)	60.5 (11.4)	58.6 (10.3)	< 0.001	< 0.001
Male, n (%)	898 (70.1)	236 (73.8)	213 (66.6)	220 (68.8)	229 (71.3)	0.216	0.494
BMI, mean (SD)	25.4 (3.6)	24.1 (3.1)	25.3 (3.6)	26.0 (4.0)	26.1 (3.3)	< 0.001	< 0.001
SBP, mean (SD), mmHg	156.7 (22.0)	154.2 (23.6)	158.3 (20.9)	155.4 (20.4)	158.8 (22.6)	0.018	0.011
DBP, mean (SD), mmHg	90.3 (13.8)	88.8 (14.1)	90.3 (13.8)	90.0 (13.8)	92.0 (13.4)	0.041	0.004
NIHSS, median (IQR)	2.0 (1.0, 3.0)	2.0 (1.0, 3.0)	2.0 (1.0, 3.0)	2.0 (1.0, 3.0)	2.0 (1.0, 3.0)	0.575	0.632
Onset time, mean (SD), hour	25.7 (19.7)	25.5 (19.4)	25.5 (20.0)	25.5 (19.9)	26.3 (19.8)	0.936	0.617
Prior mRs, n (%)						0.296	0.058
0	1063 (83.0)	254 (79.4)	268 (83.8)	264 (82.5)	277 (86.3)		
1	173 (13.5)	51 (15.9)	39 (12.2)	47 (14.7)	36 (11.2)		
2	45 (3.5)	15 (4.7)	13 (4.1)	9 (2.8)	8 (2.5)		
Smoking status, n (%)						0.634	0.37
Never	657 (52.6)	154 (49.4)	175 (55.7)	163 (51.9)	165 (53.2)		
Previous smoker	70 (5.6)	20 (6.4)	19 (6.1)	18 (5.7)	13 (4.2)		
Current smoker	523 (41.8)	138 (44.2)	120 (38.2)	133 (42.4)	132 (42.6)		
Drinking, n (%)	438 (35.0)	109 (34.7)	98 (31.1)	110 (35.4)	121 (38.7)	0.265	0.513
Previous history							
Diabetes mellitus, n (%)	387 (30.2)	31 (9.7)	65 (20.3)	103 (32.2)	188 (58.6)	< 0.001	< 0.001
Lipid disorder, n (%)	32 (2.5)	1 (0.3)	7 (2.2)	5 (1.6)	19 (5.9)	< 0.001	< 0.001
Atrial fibrillation, n (%)	8 (0.6)	4 (1.2)	3 (0.9)	1 (0.3)	0 (0)	0.103	0.062
TIA, n (%)	24 (1.9)	9 (2.8)	3 (0.9)	6 (1.9)	6 (1.9)	0.383	0.43
Ischemic stroke, n (%)	339 (26.5)	109 (34.1)	79 (24.7)	88 (27.5)	63 (19.6)	< 0.001	< 0.001
Carotid stenosis, n (%)	7 (0.5)	5 (1.6)	1 (0.3)	1 (0.3)	0 (0)	0.046	0.031
In-hospital treatment							
Antiplatelet therapy, n (%)^a^						0.827	0.497
Single antiplatelet therapy	493 (38.5)	130 (40.6)	122 (38.1)	119 (37.2)	122 (38)		
Dual antiplatelet therapy	788 (61.5)	190 (59.4)	198 (61.9)	201 (62.8)	199 (62)		
Statin therapy, n (%)^b^						0.749	0.326
Not used	43 (3.4)	7 (2.2)	13 (4.1)	10 (3.1)	13 (4)		
Normal dose	521 (40.7)	139 (43.4)	124 (38.8)	129 (40.3)	129 (40.2)		
Intensive dose	717 (56.0)	174 (54.4)	183 (57.2)	181 (56.6)	179 (55.8)		
Antihypertensive treatment	833 (67.6)	211 (67.6)	207 (68.1)	218 (70.3)	197 (64.4)	0.469	0.394
Hypoglycemic treatment	291 (22.8)	21 (6.6)	50 (15.6)	79 (24.8)	141 (44.2)	< 0.001	< 0.001
Laboratory findings							
TG, mean (SD), mg/dL	159.1 (111.5)	80.3 (19.0)	114.5 (22.6)	164.1 (42.7)	277.2 (157.4)	< 0.001	< 0.001
FBG, mean (SD), mg/dL	121.9 (51.8)	89.4 (15.5)	102.2 (19.3)	119.2 (34.5)	176.6 (67.0)	< 0.001	< 0.001
LDL-C, mean (SD), mg/dL	102.0 (32.8)	89.8 (27.9)	99.9 (28.9)	105.0 (31.0)	113.2 (38.0)	< 0.001	< 0.001
HDL-C, mean (SD), mg/dL	39.9 (12.3)	43.2 (10.6)	40.4 (10.0)	38.1 (10.2)	37.9 (16.4)	< 0.001	< 0.001
TC, mean (SD), mg/dL	164.9 (43.6)	147.0 (36.3)	158.6 (35.9)	169.0 (41.7)	184.6 (50.1)	< 0.001	< 0.001
HCY, mean (SD), mg/dL	3.3 (3.2)	3.8 (4.1)	3.3 (2.7)	3.5 (3.5)	2.7 (2.2)	< 0.001	< 0.001
PLT, mean (SD), 10^3^/µL	220.2 (66.7)	208.8 (65.4)	223.7 (67.8)	223.9 (66.0)	224.4 (66.5)	0.008	0.004
WBC, mean (SD),/µL	7183.8 (2083.5)	6758.9 (2177.6)	7088.5 (1893.5)	7364.5 (2109.0)	7512.5 (2075.8)	< 0.001	< 0.001
Uric acid, mean (SD), mg/dL	5.5 (1.6)	5.1 (1.4)	5.5 (1.6)	5.8 (1.7)	5.6 (1.7)	< 0.001	0.175
Imaging features							
Multiple infarctions, n (%)^c^	447 (34.9)	111 (34.7)	113 (35.3)	103 (32.2)	120 (37.4)	0.585	0.477
ICASB, median (IQR)^d^	1.0 (0.0, 4.0)	0.0 (0.0, 3.0)	2.0 (0.0, 4.0)	1.0 (0.0, 4.0)	2.0 (0.0, 5.0)	0.014	< 0.001
sICAS, n (%)^e^	338 (26.4)	76 (23.8)	85 (26.6)	77 (24.1)	100 (31.2)	0.122	0.036

*TyG* triglyceride-glucose, *BMI* body mass index, *SBP* systolic blood pressure, *DBP* diastolic blood pressure, *NIHSS* National Institutes of Health Stroke Scale, *mRS* modified Rankin score, *TIA* transient ischemic attack, *TG* triglyceride, *FBG* fasting blood glucose, *LDL-C* low-density lipoprotein cholesterol, *HDL-C* high-density lipoprotein cholesterol, *TC* total cholesterol, *HCY* homocysteine, *Ccr* creatinine clearance rate, *PLT* platelet, *WBC* white blood cells, *ICASB* intracranial atherosclerosis burden, *sICAS* symptomatic intracranial atherosclerosis. ^#^TyG was calculated as follows: ln (fasting TG [mg/dL] * FBG [mg/dL]/2). ^a^Single antiplatelet therapy included aspirin or clopidogrel; dual antiplatelet therapy referred to aspirin with clopidogrel. ^b^Normal dose of statin included atorvastatin 20 mg or rosuvastatin 10 mg; intensive dose of statin included atorvastatin 40–80 mg or rosuvastatin 20 mg. ^c^The presence of multiple infarctions was defined as at least two distinct lesions on DWI (separated in space or discrete on contiguous slices). ^d^ ICASB was calculated by summing the different values assigned to each intracranial artery according to the stenosis degree: 0 for no stenosis; 1 for mild stenosis; 2 for moderate stenosis; and 3 for severe stenosis or occlusion. ^e^The presence of sICAS was determined by a combination of clinical manifestations, the location of the infarct lesion, and the corresponding artery with moderate-to-severe stenosis. SI conversion factors: To convert cholesterol to millimoles per liter, multiply by 0.0259; FBG to microkatals per liter, 0.0555; TG to millimoles per liter, 0.0113; HCY to micromoles per liter, 7.397; WBC to count per liter, 0.001; and uric acid to millimoles per liter, 0.0595. p*: ANOVA test among the four groups. p**: comparison between TyG quartile 4 vs. TyG quartile 1

Among patients with sICAS, the M1 and M2 segments were the most represented responsible arteries (Additional file 1: Fig. S2). In addition, there was no significant difference in TyG levels among the different types of responsible artery groups (Additional file 1: Fig. S3).

### Regression analysis of TyG with sICAS and ICASB

**Fig. 2:**
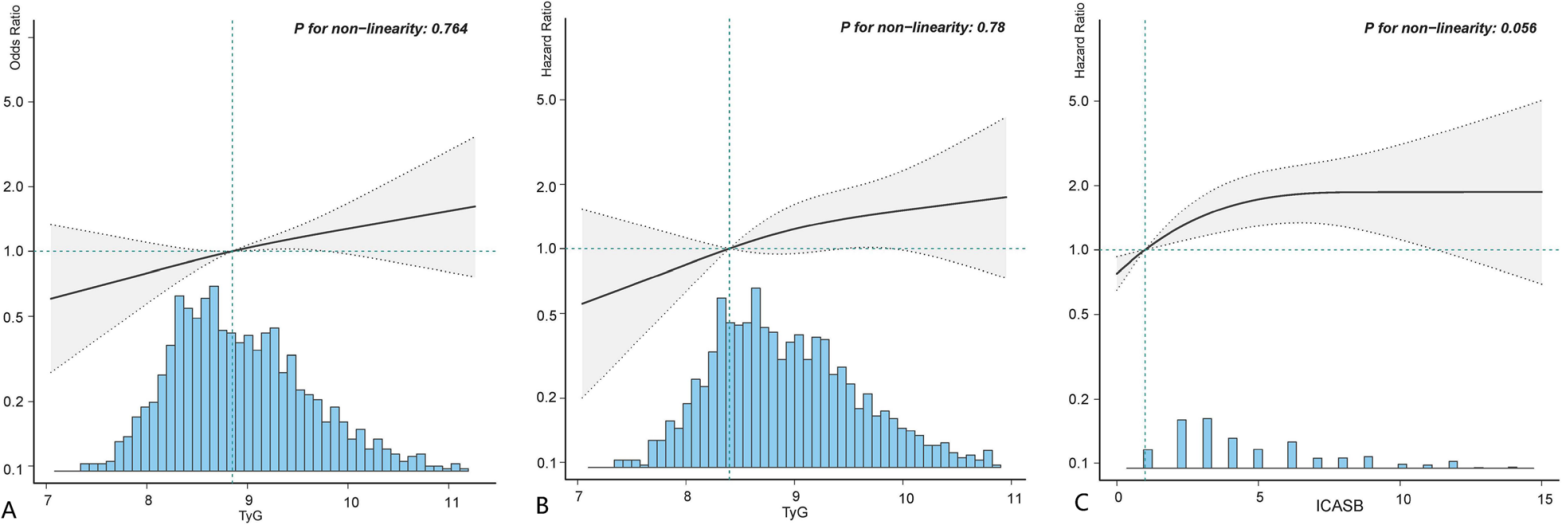
The dose‒response relationship between TyG and sICAS, TyG and stroke recurrence, and ICASB and stroke recurrence. The solid line indicates the estimated hazard ratio, and the dashed lines indicates the 95% confidence intervals. **A** relationship between TyG and sICAS; **B** relationship between TyG and stroke recurrence; **C** relationship between ICASB and stroke recurrence. A model with three knots located at the 5th, 50th, and 95th percentiles was applied

The study first investigated the relationship between elevated TyG levels and the prevalence of sICAS, as well as the effect of TyG on the ICAS burden. Initially, univariate logistic regression analysis suggested that female, NIHSS at arrival, LDL-C, TC, WBC, TyG, ICASB and the presence of multiple infarctions were associated with sICAS (Additional file 1: Table S4). A multivariate logistic regression analysis was performed between TyG and sICAS, adjusting for potential confounders selected based on the results of univariate analysis and literature reports (Table 2). The results showed that the risk of sICAS increased by 25% for each unit increase in TyG when considered as a continuous variable (OR 1.25 95% CI 1.01–1.55, p = 0.037). When TyG was considered as quartiles, there was a trend of increasing risk of sICAS with elevated TyG levels, and the risk increased by 59% in the fourth quartile of TyG compared with the first quartile (OR 1.59, 95% CI 1.04–2.43, p = 0.033), with the first quartile set as the reference group. The dose‒response relationship between TyG and the incidence of sICAS was explored by RCS. A linear dose‒response relationship between TyG and sICAS was found (Fig. 2A). The study also analyzed the linear regression relationship between TyG and ICASB (Table 2), adjusting for covariates selected based on the results of univariate analysis (Additional file 1: Table S5) and literature reports. The results showed that higher TyG was associated with increased ICASB (β 0.43, 95% CI 0.13–0.74, p = 0.006), when considered as a continuous variable.. There was also a significant increase in ICASB when TyG was considered as quartiles. When TyG was considered as quartiles, the fourth quartile had ICASB increased by 1.08 (β 1.08, 95% CI 0.47–1.69, p = 0.001), with the first quartile set as the reference group. In addition, contour plots were used to explore whether the effect of TyG on sICAS was consistent across people of different ages, BMI, SBP and DBP on admission. The risk of sICAS tended to increase more dramatically with TyG in people with higher age, BMI, SBP and DBP (Additional file 1: Fig. S4). In the sICAS subgroup, the association between the TyG index and the degree of sICAS (50% to 69% as moderate, 70 to 99% as considered severe and occlusion) was analyzed. Patients in the fourth quartile of TyG had a higher risk of severe or occluded sICAS (OR 2.26, 95% CI 1.02–5.04, p = 0.046), with the first quartile of TyG set as the reference group (Additional file 1: Table S6).

**Table 2:** Relationship of TyG with sICAS and ICASB

	TyG quantiles^*^	OR/β (95% CI) (TyG as quantiles)	P value	OR/β (95% CI) (TyG as continuous)	P value
sICAS^a^					
Unadjusted OR (95%CI)	Q1 (< 8.4)	1 (Ref.)		1.19 (1–1.41)	0.048
	Q2 (8.4–8.9)	1.16 (0.81–1.66)	0.412		
	Q3 (8.9–9.4)	1.02 (0.71–1.46)	0.926		
	Q4 (≥ 9.4)	1.45 (1.02–2.06)	0.036		
	P for trend		0.074		
Adjusted model I OR (95%CI)	Q1 (< 8.4)	1 (Ref.)		1.23 (1.03–1.46)	0.022
	Q2 (8.4–8.9)	1.16 (0.81–1.67)	0.411		
	Q3 (8.9–9.4)	1.05 (0.73–1.52)	0.799		
	Q4 (≥ 9.4)	1.54 (1.08–2.2)	0.018		
	P for trend		0.036		
Adjusted model II OR (95%CI)	Q1 (< 8.4)	1 (Ref.)		1.25 (1.01–1.55)	0.037
	Q2 (8.4–8.9)	1.16 (0.79–1.71)	0.445		
	Q3 (8.9–9.4)	1.02 (0.68–1.53)	0.917		
	Q4 (≥ 9.4)	1.59 (1.04–2.43)	0.033		
	P for trend		0.074		
ICASB^b^					
Unadjusted β (95%CI)	Q1 (< 8.4)	0 (Ref.)		0.29 (0.03, 0.55)	0.03
	Q2 (8.4–8.9)	0.63 (0.1, 1.16)	0.02		
	Q3 (8.9–9.4)	0.52 (− 0.01, 1.05)	0.055		
	Q4 (≥ 9.4)	0.81 (0.27, 1.34)	0.003		
	P for trend		0.007		
Adjusted model I β (95%CI)	Q1 (< 8.4)	0 (Ref.)		0.46 (0.2–0.72)	0.001
	Q2 (8.4–8.9)	0.72 (0.2–1.25)	0.007		
	Q3 (8.9–9.4)	0.76 (0.23–1.28)	0.005		
	Q4 (≥ 9.4)	1.15 (0.62–1.68)	< 0.001		
	P for trend		< 0.001		
Adjusted model II β (95%CI)	Q1 (< 8.4)	0 (Ref.)		0.43 (0.13–0.74)	0.006
	Q2 (8.4–8.9)	0.74 (0.19–1.28)	0.008		
	Q3 (8.9–9.4)	0.63 (0.07–1.19)	0.028		
	Q4 (≥ 9.4)	1.08 (0.47–1.69)	0.001		
	P for trend		0.002		

*The first quantile of TyG was set as the reference group. ^a^The relationship between TyG and sICAS was examined by univariate and multivariate logistic regression models. ^b^The relationship between TyG and ICASB (as continuous) was examined by univariate and multivariate linear regression models. Model I was adjusted for age and sex, and model II was adjusted for BMI, SBP, DBP, smoking status, drinking, previous stroke, and previous diabetes mellitus. Confounders were determined based on univariate analysis and previous literature reports

### Effect of TyG, sICAS, and ICASB on stroke recurrence

During the 90-day follow-up, a total of 17 patients were lost to follow-up, and 117 patients experienced recurrent ischemic stroke. Univariate Cox regression analysis suggested that SBP, time after onset, previous history of diabetes mellitus, lipid disorder, FBG, WBC, TyG as quantiles, ICASB, sICAS, and presence of multiple infarctions were risk factors for stroke recurrence (Additional file 1: Table S7). Table 3 demonstrates the relationship between TyG, ICASB, sICAS, and ischemic stroke recurrence after adjusting for covariates. The fourth quartile of TyG independently increased the risk of stroke recurrence (HR 2.02, 95% CI 1.07–3.84, p = 0.025), with the first quartile of TyG set as the reference group. The presence of sICAS was associated with a 1.65-fold increased risk of recurrence (HR 1.65, 95% CI 1.1–2.46, p = 0.014). Additionally, ICASB was also significantly associated with the risk of recurrence (HR 1.08 as continuous, 95% CI 1.03–1.13, p = 0.002), and patients with ICASB > 5 had a higher risk of recurrence (HR 1.76 as categorial, 95% CI 1.1–2.81, p = 0.018, with ICASB < 4 as reference group). A sensitivity analysis was performed after multiple imputation of missing data, and the main results were still robust (Additional file 1: Table S8). RCS plots were used to assess the dose‒response relationship between TyG, ICASB and stroke recurrence. Similar linear dose‒response relationships between TyG and stroke recurrence and between ICASB and stroke recurrence were found. (Fig. 2B, C).

**Table 3:** Association of TyG, sICAS, and ICASB with ischemic stroke recurrence

Variable	No. total	No. event (%)	Unadjusted HR (95% CI)	P value	Model I HR (95% CI)	P value	Model II HR (95% CI)	P value	Model III HR (95% CI)	P value
TyG (continuous)	1281	117 (9.1)	1.15 (0.9–1.47)	0.254	1.12 (0.86–1.46)	0.388	1.04 (0.78–1.38)	0.812	1.13 (0.85–1.49)	0.403
TyG (categories)										
TyG quartile 1	320	19 (5.9)	1 (Ref.)		1 (Ref.)		1 (Ref.)		1 (Ref.)	
TyG quartile 2	320	32 (10)	1.73 (0.98–3.05)	0.059	1.85 (1.02–3.36)	0.043	1.82 (0.97–3.41)	0.061	1.72 (0.91–3.26)	0.065
TyG quartile 3	320	32 (10)	1.72 (0.98–3.04)	0.061	1.85 (1.01–3.38)	0.046	1.88 (1.01–3.53)	0.048	1.96 (1.05–3.66)	0.043 0.010
TyG quartile 4	321	34 (10.6)	1.84 (1.05–3.23)	0.033	1.9 (1.04–3.48)	0.038	1.91 (1.12–3.15)	0.027	2.02 (1.07–3.84)	0.025
ICASB (continuous)	1281	117 (9.1)	1.07 (1.03–1.12)	0.001	1.08 (1.04–1.13)	< 0.001	1.08 (1.03–1.13)	0.003	1.08 (1.03–1.13)	0.002
ICASB (categories)										
ICASB < 4	922	70 (7.6)	1 (Ref.)		1 (Ref)		1 (Ref.)		1 (Ref.)	
ICASB 4–5	137	17 (12.4)	1.67 (0.98–2.84)	0.058	1.84 (1.07–3.14)	0.026	1.57 (0.88–2.79)	0.125	1.73 (1–2.99)	0.05
ICASB > 5	222	30 (13.5)	1.8 (1.18–2.76)	0.007	1.86 (1.18–2.92)	0.007	1.82 (1.14–2.92)	0.012	1.76 (1.1–2.81)	0.018
sICAS										
Without sICAS	943	70 (7.4)	1 (Ref.)		1 (Ref.)		1 (Ref.)		1 (Ref.)	
With sICAS	338	47 (13.9)	1.89 (1.31–2.74)	0.001	1.94 (1.32–2.84)	0.001	1.73 (1.16–2.59)	0.007	1.65 (1.1–2.46)	0.014

The effects of TyG, sICAS, and ICASB on ischemic stroke recurrence were analyzed using univariate and multifactorial Cox regression models. Model I was adjusted for age and sex, and model II was further adjusted for BMI, SBP, smoking status, time at onset, NIHSS score at arrival, WBC count, previous stroke, and previous diabetes mellitus. Model III was further adjusted for treatment during admission (antiplatelet and intensive statin therapy) and antihypertensive and hypoglycemic treatment on the basis of model II

**Fig. 3:**
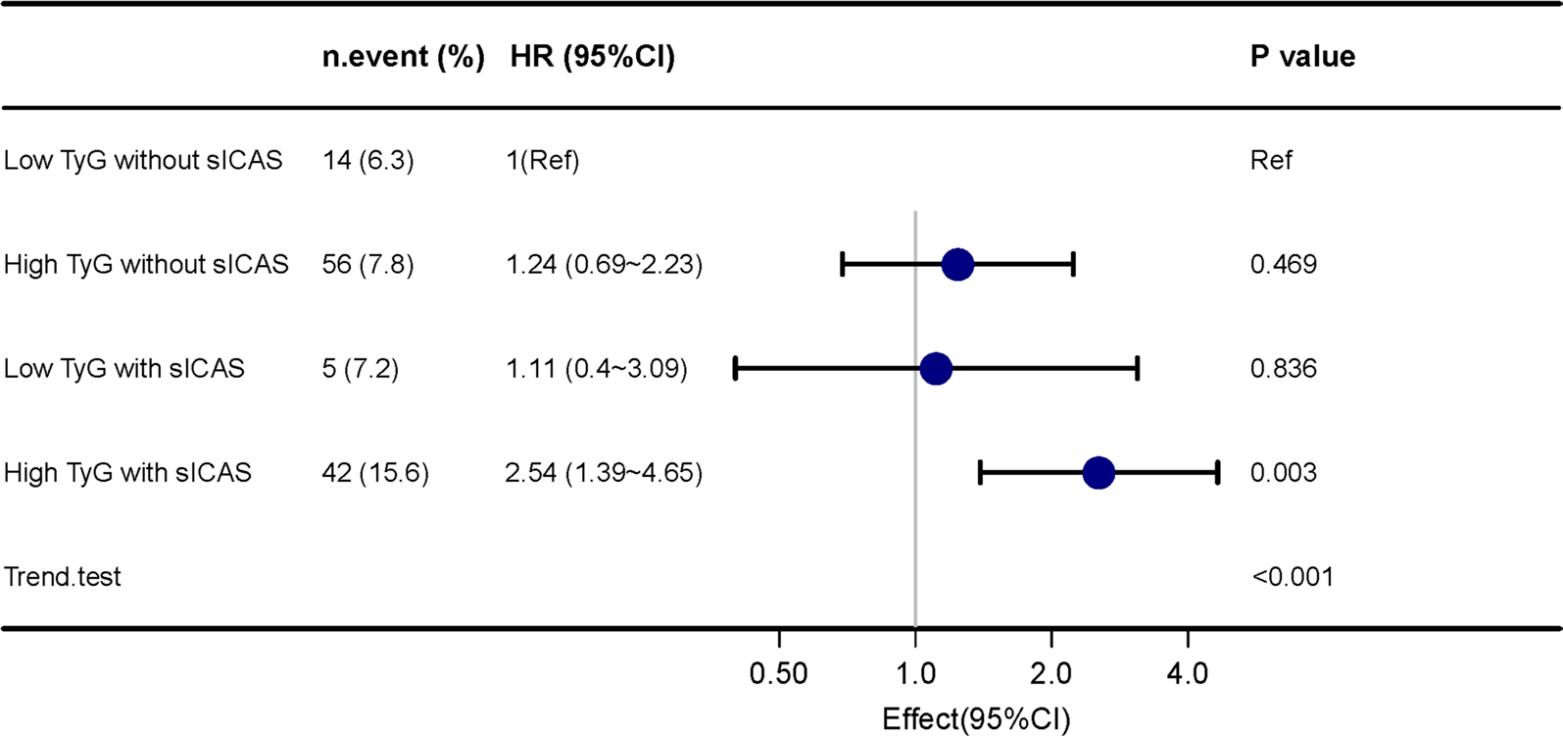
Synergistic effect of elevated TyG and presence of sICAS on ischemic stroke recurrence. Patients were dichotomized into low TyG group and high TyG group (low TyG < 8.4 versus high TyG ≥ 8.4). The cutoff point of TyG was determined by X-tile. Patients with high TyG and the presence of sICAS had the highest risk of recurrence

**Fig. 4:**
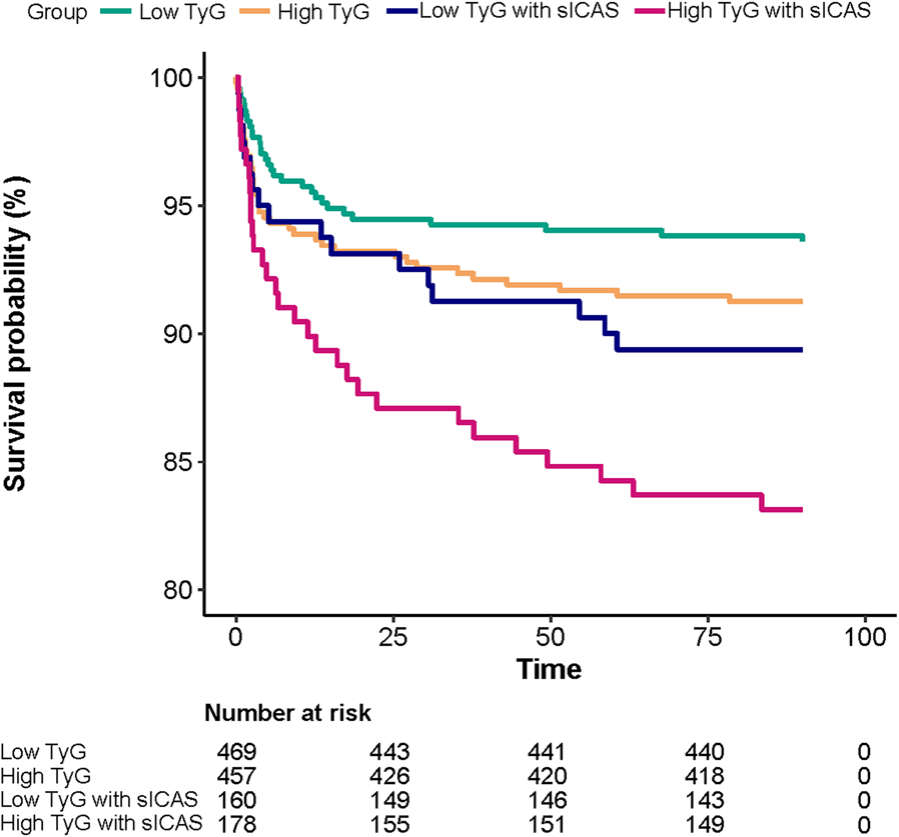
Survival curves in patients with or without sICAS at low or high TyG levels. Green, orange, blue and red curves indicate the survival status of patients with low TyG without presence of sICAS, high TyG without presence of sICAS, low TyG with presence of sICAS, and high TyG with presence of sICAS, respectively

To further clarify whether there was a synergistic effect of TyG and sICAS on stroke recurrence, patients were dichotomized into low TyG group and high TyG group (low TyG < 8.4 versus high TyG ≥ 8.4). The cutoff point of TyG was determined by X-tile (Additional file 1: Fig. S5). According to TyG dichotomous grouping and the presence of sICAS, patients were divided into four groups: low TyG without sICAS, high TyG without sICAS, low TyG with sICAS and high TyG with sICAS. Multivariate Cox regression showed the highest risk of recurrence in patients with high TyG and the presence of sICAS (HR 2.54, 95% CI 1.39–4.65, p = 0.003) when patients with low TyG and without sICAS were set as the reference group (Fig. 3). The same trend was observed as the burden of ICAS increased (Additional file 1: Fig. S6). The presence of sICAS and higher ICASB jointly increased the risk of recurrence. A significant interaction effect of TyG with sICAS was found (P for interaction = 0.043). In the sICAS group, high TyG was associated with a 3.09-fold increase in stroke recurrence compared to low TyG. However, the effect was not observed in the non-sICAS group (HR, 1.23; 95% CI, 0.66–2.3, P = 0.519) (Additional file 1: Table S9). Survival curves suggested that recurrent strokes were concentrated in the first 2 weeks after onset (Fig. 4). Finally, the IDI and NRI results showed that the addition of TyG can improve the differentiation and reclassification beyond traditional risk factors (age, sex, BMI, SBP, smoking status, time at onset, NIHSS score at arrival, WBC count, previous stroke, previous diabetes mellitus and in-hospital treatment including antiplatelet, antihypertensive, hypoglycemic and statin therapy), with a continuous NRI of 0.188 (95% CI: 0.0066–0.268) and an IDI of 0.01 (95% CI: 0.003–0.03) (Additional file 1: Table S10 and Fig. S7).

## Discussion

Our study confirmed that in acute minor stroke patients with hypertension, higher TyG reflected an increased burden of ICAS and risk of sICAS. This relationship was more prominent in those with older age and higher admission blood pressure and BMI. Our results also suggested a synergistic effect of high TyG levels and the presence of sICAS on stroke recurrence in minor stroke patients with hypertension.

### Relationship between IR and ICAS in the hypertensive population

Atherosclerosis is a serious vascular pathological change in the hypertensive population. Clinical studies have found that hypertension is a major risk factor for developing arterial stiffness and atherosclerosis [22]. In addition, vascular stiffness and luminal narrowing can cause secondary hypertension. Hypertension and atherosclerosis are mutually influential and aggravating [23]. The mechanism of atherosclerosis caused by hypertension may be due to the elevated pressure on the vessel wall causing retraction and continuity damage to endothelial tissues, leading to mechanical injury and abnormal function of the endothelium [6].At the same time, hypertension is often combined with abnormal lipid metabolism [24]. Abnormal lipid metabolism, such as high triglycerides and LDL-C, can accumulate in damaged endothelial tissue, causing fibrosis of the endothelium, stiffening of the vessel wall and narrowing of the lumen, ultimately leading to ischemic changes in the supplied organs [25]. Moreover, abnormal metabolic status, such as hyperlipidemia and hyperglycemia, significantly increases the risk of atherosclerotic disease in people with hypertension, in which IR plays a key role [26]. IR contributes to the development of lipid disorders [27] and accelerates the production of connective tissue in the vessel wall and the aggregation of LDL-C into the arterial smooth muscle [28]. Additionally, overproduction of insulin increases the inflammatory response at the endothelium and reduces the synthesis and utilization of nitric oxide in endothelial cells, affecting the normal function of the endothelium [29]. IR produces more reactive oxygen species and induces an abnormal increase in cytokines and inflammatory factors, leading to lipid peroxidation and endothelial damage and ultimately promoting the formation of atherosclerosis [30]. In addition, long-term hyperinsulinemia leads to an increase in baseline blood pressure, and IR and hypertension together promote the development of atherosclerosis [7].

In the past, the relationship between IR and ICAS has been confirmed. A cohort study demonstrated that IR is an independent risk factor for moderate to severe large artery atherosclerotic stroke [4]. Another case‒control study found that ischemic stroke patients had higher homeostasis model assessment of insulin resistance (HOMA-IR) levels than controls in both the acute phase and 3-month follow-up of stroke onset, and HOMA-IR was associated with the severity of stroke and worse prognosis [31]. However, little attention has been given to the relationship between IR and atherosclerosis in patients with hypertension, who may endure more from IR and atherosclerosis, except for the study by Wu et al., which revealed that TyG, as a reliable IR biomarker, may increase the risk and severity of atherosclerosis in hypertensive individuals [32]. Furthermore, evidence in ICAS or sICAS patients with hypertension is also scarce. Our study supplemented the evidence that in hypertensive patients, TyG is linearly associated with the burden of ICAS, as measured by the number and degree of stenosis to the intracranial arteries. We also found that increased TyG may reflect a higher risk of sICAS incidence and more severe and occluded sICAS in hypertensive patients.

TyG, a simple and readily available measurement of IR, has gained increasing attention due to its ability to evaluate arterial stiffness and the risk of cardiovascular and cerebrovascular diseases [32]. However, previous studies have primarily focused on traditional risk factors, such as hypertension, diabetes, and hyperlipidemia, and have not extensively explored the relationship between TyG and ICAS severity [26]. Our study provides preliminary evidence that TyG can serve as a novel risk factor for determining the severity of ICAS and the occurrence of sICAS in hypertensive patients. For sICAS patients who have not received timely and comprehensive evaluation of intracranial vascular morphology, TyG can be used to preliminarily estimate the risk of severe stenosis or occlusion, alerting clinicians to pay early attention and provide timely intervention.

In addition, we found that the role of TyG in promoting sICAS was more pronounced in the older population, likely due to age-related arterial intima-media thickening [33]. With age, arterial smooth muscle can proliferate into the intima and produce fibrous connective tissue, thickening the intima and increasing the risk of atherosclerosis [34]. Furthermore, increased blood pressure and blood pressure variability can indicate endothelial damage and increased arterial stiffness [35], which may explain why the predictive effect of TyG on sICAS was stronger in individuals with higher blood pressure. BMI is another key predictor of atherosclerosis. Previous studies have confirmed BMI is associated with increased arterial stiffness and atherosclerosis [36, 37]. However, the obesity paradox has been proposed in the past, with findings of less aortic atherosclerosis in some autopsies with morbid obesity [38]. There is limited evidence on the association between BMI and sICAS. Our study tentatively confirms that BMI is a potential risk factor for sICAS, pending further confirmation in future large cohorts.

### Possible mechanisms of IR leading to stroke recurrence

The present study also confirmed that higher TyG is associated with short-term recurrence in minor stroke patients, which is consistent with previous studies. Miao et al. demonstrated that stroke patients with a higher TyG index were associated with worse neurological prognosis and a higher risk of death [12]. In addition, TyG is also associated with early neurological deterioration and long-term stroke recurrence [39, 40]. Although traditional vascular risk factors such as hyperglycemia and dyslipidemia are believed to contribute to stroke incidence and poor prognosis, recent studies have found that even with aggressive interventions for traditional risk factors, such as intensive lipid lowering and glucose lowering, a residual risk of cardiovascular events still cannot be eliminated [41]. A Mendelian randomized study confirmed that the IR-specific genotype is associated with an increased risk of coronary artery disease, myocardial infarction, ischemic stroke, and small artery occlusion subtype of stroke [42]. A randomized controlled trial published in the New England Journal of Medicine in 2016 showed that pioglitazone, which improves IR, could reduce the risk of stroke recurrence [43]. These studies provide ample evidence that IR may play a key role in the prognosis of ischemic stroke. Similarly, in our study, by calculating the IDI and NRI, we found that TyG had incremental discrimination value of stroke recurrence beyond the traditional factors. This suggests that improving IR is crucial for preventing stroke recurrence in clinical practice.

The mechanisms may include the following: First, IR can affect platelet activation, adhesion and aggregation, causing thrombosis [40]. Second, IR increases the risk of subclinical atherosclerosis and reduces plaque stability by inducing chronic inflammation, endothelial dysfunction and foam cell formation [28, 44]. People with rupture-prone plaques are more likely to experience short-term ischemic stroke recurrence.

### IR causes stroke recurrence mainly through involvement in ICAS

Our study further confirms that IR and ICAS have a synergistic effect on increasing the risk of short-term stroke recurrence. The initial hypothesis was derived from a cardiology research that showed a significant association between IR and the degree of coronary stenosis and the risk of cardiovascular events [45], and severe coronary artery stenosis combined with IR could further increase the likelihood of ischemic events [46]. IR is involved in the development of pan-vascular lesions and ultimately leads to clinical diseases [47]. To demonstrate that the relationship is also present in stroke patients, we introduced a method to assess the burden of ICAS and explored the joint effect of TyG and ICASB or sICAS on stroke prognosis. The results confirmed that patients with high TyG combined with sICAS endured a higher risk of recurrence. Our findings implied that the interaction effect between TyG and the existence of sICAS leads to a high risk of recurrence. These findings suggest that monitoring the TyG index in sICAS patients is crucial, and medications that improve IR can be used to prevent stroke recurrence in these patients.

This synergistic effect may be due to several reasons. First, higher IR levels in the presence of sICAS exacerbate stenosis progression in the acute phase, further decreasing blood flow and increasing the risk of early infarct progression [48]. Secondly, TyG may increase the chance of fibrous cap rupture, intraplaque hemorrhage of unstable plaques and thrombosis in other arteries with preexisting stenosis, resulting in new infarctions [49, 50]. A similar synergistic effect was also observed between TyG and ICASB. This is probably because ICASB reflects a higher proportion of moderate to severe stenosis with more arteries involved, making the risk of multiple infarcts in the acute phase significantly higher. Thus, patients with a heavier ICAS burden and elevated TyG were exposed to a higher risk of new infarctions.

### Research strengths and limitations

The strengths of the study can be summarized as follows: Firstly, the study confirmed for the first time that TyG was associated with sICAS and ICAS burden. Secondly, to strengthen the reliability of the findings, potential covariates were adjusted, and the TyG index was treated as continuous and categorical variables. Thirdly, the prospective cohort design strengthens the study's findings that combining TyG and imaging assessment of ICAS can effectively predict the short-term risk of stroke recurrence. Lastly, the study highlights the importance of considering ICAS as a systemic disease that requires early intervention and treatment, with a focus on improving insulin resistance.

However, the present study also has some limitations. First, it remains to be assessed whether the downstream biological mechanisms of IR, such as platelet activation, chronic inflammation, endothelial dysfunction, and foam cell formation, mediate the relationship between TyG and ICAS. Second, due to the original design of the cohort, there was a lack of follow-up of the incidence of sICAS, and the causal relationship between TYG and sICAS cannot be adequately demonstrated. In addition, the study did not perform a dynamic assessment of repeated TyG measurements, which may further clarify the long-term impact of TyG on the development of ICAS.

## Conclusions

For acute minor stroke patients with preexisting hypertension, TyG, as a reliable marker of IR, was associated with an increased burden of ICAS and risk of sICAS. This relationship was particularly pronounced in patients with older age, higher admission blood pressure and BMI. Furthermore, there was a synergistic effect between elevated TyG and sICAS, which increased the risk of stroke recurrence within 90 days in minor stroke patients with hypertension.

## Supplementary Information

**Additional file 1: Table S1**. Frequency and percentage of missing variables. **Table S2.** Baseline characteristics of patients included and excluded due to missing data of TG, FBG and intracranial artery imaging. **Table S3.** Baseline characteristics of patients with or without sICAS. **Table S4.** Univariate logistic regression analysis of risk factors associated with sICAS. **Table S5.** Univariate linear regression analysis of risk factors associated with ICASB. **Table S6.** Subgroup analysis between TyG index and the degree of sICAS**. Table S7.** Univariate Cox regression analysis of risk factors associated with ischemic stroke recurrence. **Table S8.** Sensitivity analysis after multiple imputation of variates with missing values. **Table S9.** Interaction effect on stroke recurrence between TyG and sICAS**. Table S10.** The value of TyG improved the risk stratification of stroke recurrence according to continuous-NRI and IDI**. Figure S1.** Pattern of missing data. **Figure S2.** Distribution of arteries responsible for sICAS. **Figure S3.** Association between responsible sICAS arteries and TyG. **Figure S4.** Contour plots reflecting the effect of age, BMI, and blood pressure on the relationship between TyG and sICAS. **Figure S5.** X-tile analyses of TyG optimal cutoff value. **Figure S6.** Combined effect of TyG and burden of ICAS on ischemic stroke recurrence. **Figure S7.** IDI plot.

## Data Availability

The raw data supporting the conclusions of this article will be made available upon appropriate request.

## Abbreviations

TyG: Triglyceride-glucose
ICAS: Intracranial atherosclerosis
sICAS: Symptomatic intracranial atherosclerosis
FBG: Fasting blood glucose
TG: Triglyceride
RCS: Restricted cubic spline
IR: Insulin resistance
SEACOAST: Safety and efficacy of aspirin-clopidogrel in acute noncardiogenic minor ischemic stroke: a prospective and multicenter study based on the real world
NIHSS: National Institutes of Health Stroke Scale
MRA: Magnetic resonance angiography
CTA: Computed tomographic arteriography
DSA: Digital subtraction angiography
mRS: Modified Rankin score
TIA: Transient ischemic attack
CT: Computed tomography
MRI: Magnetic resonance imaging
BMI: Body mass index
SBP: Systolic blood pressure
DBP: Diastolic blood pressure
LDL-C: Low-density lipoprotein cholesterol
HDL-C: High-density lipoprotein cholesterol
TC: Total cholesterol
HCY: Homocysteine
PLT: Platelet
WBC: White blood cells
DWI: Diffusion-weighted imaging
ICA: Internal carotid artery
ACA: Anterior cerebral artery
PCA: Posterior cerebral artery
VA: Vertebral artery
BA: Basilar artery
WASID: Warfarin Aspirin Symptomatic Intracranial Disease
ICASB: Intracranial atherosclerosis burden
IDI: Integrated discrimination improvement, NRI, net reclassification improvement
HOMA-IR: Homeostasis model assessment of insulin resistance
