# Supplementary material for: Triglyceride-glucose index, symptomatic intracranial artery stenosis and recurrence risk in minor stroke patients with hypertension
Source: Cardiovasc Diabetol. 2023 Apr 19;22:90. doi: 10.1186/s12933-023-01823-6 (PMC10114394; doi:10.1186/s12933-023-01823-6)
Supplement: Supplementary file 1 — Additional file 1: Table S1. Frequency and percentage of missing variables. Table S2. Baseline characteristics of patients included and excluded due to missing data of TG, FBG and intracranial artery imaging. Table S3. Baseline characteristics of patients with or without sICAS. Table S4. Univariate logistic regression analysis of risk factors associated with sICAS. Table S5. Univariate linear regression analysis of risk factors associated with ICASB. Table S6. Subgroup analysis between TyG index and the degree of sICAS. Table S7. Univariate Cox regression analysis of risk factors associated with ischemic stroke recurrence. Table S8. Sensitivity analysis after multiple imputation of variates with missing values. Table S9. Interaction effect on stroke recurrence between TyG and sICAS. Table S10. The value of TyG improved the risk stratification of stroke recurrence according to continuous-NRI and IDI. Figure S1. Pattern of missing data. Figure S2. Distribution of arteries responsible for sICAS. Figure S3. Association between responsible sICAS arteries and TyG. Figure S4. Contour plots reflecting the effect of age, BMI, and blood pressure on the relationship between TyG and sICAS. Figure S5. X-tile analyses of TyG optimal cutoff value. Figure S6. Combined effect of TyG and burden of ICAS on ischemic stroke recurrence. Figure S7. IDI plot. [file 12933_2023_1823_MOESM1_ESM.docx]

**Additional file 1**

Table S1 Frequency and percentage of missing variables.

Table S2 Baseline characteristics of patients included and excluded due to missing data of TG, FBG and intracranial artery imaging.

Table S3 Baseline characteristics of patients with or without sICAS.

Table S4 Univariate logistic regression analysis of risk factors associated with sICAS.

Table S5 Univariate linear regression analysis of risk factors associated with ICASB.

Table S6 Subgroup analysis between TyG index and the degree of sICAS

Table S7 Univariate Cox regression analysis of risk factors associated with ischemic stroke recurrence.

Table S8 Sensitivity analysis after multiple imputation of variates with missing values.

Table S9 Interaction effect on stroke recurrence between TyG and sICAS

Table S10 The value of TyG improved the risk stratification of stroke recurrence according to continuous-NRI and IDI

Figure S1 Pattern of missing data.

Figure S2 Distribution of arteries responsible for sICAS.

Figure S3 Association between responsible sICAS arteries and TyG.

Figure S4 Contour plots reflecting the effect of age, BMI, and blood pressure on the relationship between TyG and sICAS.

Figure S5 X tile analyses of TyG optimal cut-off value.

Figure S6 Combined effect of TyG and burden of ICAS on ischemic stroke recurrence.

Figure S7 IDI plot.

**Table S1 Frequency and percentage of missing variables.**

| **Variable** | **Frequency** | **Percentage, %** |
| --- | --- | --- |
| WBC | 78 | 6.089 |
| BMI | 76 | 5.9329 |
| PLT | 58 | 4.5277 |
| Smoking | 31 | 2.42 |
| Drinking | 28 | 2.1858 |
| HCY | 22 | 1.7174 |
| Follow up | 17 | 1.3271 |
| HDL | 9 | 0.7026 |
| SBP | 4 | 0.3123 |
| DBP | 4 | 0.3123 |
| LDL | 3 | 0.2342 |
| TC | 1 | 0.0781 |
| Age | 0 | 0 |
| NIHSS | 0 | 0 |
| Gender | 0 | 0 |
| Onset time | 0 | 0 |
| Diabetes | 0 | 0 |
| Lipid disorder | 0 | 0 |
| Past mRs | 0 | 0 |
| ICAS | 0 | 0 |
| Atrial fibrillation | 0 | 0 |
| Previous ischemic stroke | 0 | 0 |
| Previous TIA | 0 | 0 |
| Carotid stenosis | 0 | 0 |

**Table S2 Baseline characteristics of patients included and excluded due to missing data of TG, FBG and intracranial artery imaging.**

| **Variables** | **Total (n = 1854)** | **Excluded (n = 573)** | **Included (n = 1281)** | **p** |
| --- | --- | --- | --- | --- |
| Age, mean (SD), year | 62.1 (11.6) | 63.3 (11.5) | 61.6 (11.6) | 0.005 |
| Gender, n (%) |  |  |  | 0.899 |
| Female | 556 (30.0) | 173 (30.2) | 383 (29.9) |  |
| Male | 1298 (70.0) | 400 (69.8) | 898 (70.1) |  |
| BMI, mean (SD) | 25.2 (3.5) | 25.0 (3.4) | 25.4 (3.6) | 0.041 |
| SBP, mean (SD), mmHg | 156.4 (21.7) | 155.8 (21.1) | 156.7 (22.0) | 0.435 |
| DBP, mean (SD), mmHg | 90.1 (13.9) | 89.6 (14.1) | 90.3 (13.8) | 0.315 |
| NIHSS, Median (IQR) | 2.0 (1.0, 3.0) | 2.0 (1.0, 3.0) | 2.0 (1.0, 3.0) | 0.85 |
| Onset time, mean (SD), hour | 25.5 (19.8) | 24.9 (20.0) | 25.7 (19.7) | 0.448 |
| Prior mRs, n (%) |  |  |  | 0.007 |
| 0 | 1507 (81.3) | 444 (77.5) | 1063 (83) |  |
| 1 | 283 (15.3) | 110 (19.2) | 173 (13.5) |  |
| 2 | 64 ( 3.5) | 19 (3.3) | 45 (3.5) |  |
| Smoking status, n (%) |  |  |  | 0.022 |
| Never | 952 (52.8) | 295 (53.2) | 657 (52.6) |  |
| Previous smoker | 119 ( 6.6) | 49 (8.8) | 70 (5.6) |  |
| Current smoker | 733 (40.6) | 210 (37.9) | 523 (41.8) |  |
| Drinking, n (%) | 616 (34.0) | 178 (32) | 438 (35) | < 0.001 |
| Diabetes mellitus, n (%) | 556 (30.0) | 169 (29.5) | 387 (30.2) | 0.756 |
| Lipid disorder, n (%) | 47 ( 2.5) | 15 (2.6) | 32 (2.5) | 0.88 |
| Atrial fibrillation, n (%) | 11 ( 0.6) | 3 (0.5) | 8 (0.6) | 1 |
| TIA, n (%) | 30 ( 1.6) | 6 (1) | 24 (1.9) | 0.192 |
| Ischemic stroke, n (%) | 506 (27.3) | 167 (29.1) | 339 (26.5) | 0.231 |
| Carotid stenosis, n (%) | 8 ( 0.4) | 1 (0.2) | 7 (0.5) | 0.448 |
| Antiplatelet therapy, n (%) |  |  |  | 0.354 |
| Single antiplatelet therapy | 724 (39.1) | 233 (40.7) | 491 (38.4) |  |
| Dual antiplatelet therapy | 1128 (60.9) | 340 (59.3) | 788 (61.6) |  |
| Statin therapy, n (%) |  |  |  | 0.014 |
| Not used | 55 ( 3.0) | 12 (2.1) | 43 (3.4) |  |
| Normal dose | 722 (38.9) | 201 (35.1) | 521 (40.7) |  |
| Intensive dose | 1077 (58.1) | 360 (62.8) | 717 (56) |  |
| LDL-C, mean (SD), mg/dL | 101.0 (33.0) | 98.3 (33.4) | 102.0 (32.8) | 0.035 |
| HDL-C, mean (SD), mg/dL | 39.7 (11.6) | 39.2 (9.6) | 39.9 (12.3) | 0.259 |
| TC, mean (SD), mg/dL | 162.4 (43.5) | 156.4 (42.9) | 164.9 (43.6) | < 0.001 |
| HCY, mean (SD), mg/dL | 3.2 (3.0) | 2.9 (2.3) | 3.3 (3.2) | 0.015 |
| PLT, mean (SD), 10^3^/µL | 219.4 (65.3) | 217.5 (61.8) | 220.2 (66.7) | 0.429 |
| WBC, mean (SD), /µL | 7251.8 (2193.5) | 7422.1 (2441.6) | 7183.8 (2083.5) | 0.044 |
| Uric acid, mean (SD), mg/dL | 5.5 (1.6) | 5.4 (1.5) | 5.5 (1.6) | 0.138 |

**Table S3 Baseline characteristics of patients with or without sICAS.**

| **Variables** | **Total (n = 1281)** | **Without sICAS (n = 943)** | **With sICAS (n = 338)** | **p** |
| --- | --- | --- | --- | --- |
| Age, mean (SD) | 61.6 (11.6) | 61.3 (11.5) | 62.6 (11.8) | 0.067 |
| Male, n (%) | 898 (70.1) | 676 (71.7) | 222 (65.7) | 0.039 |
| BMI, mean (SD) | 25.4 (3.6) | 25.3 (3.4) | 25.5 (4.1) | 0.437 |
| Systolic pressure, mean (SD) | 156.7 (22.0) | 156.2 (21.8) | 158.0 (22.6) | 0.21 |
| Diastolic pressure, mean (SD) | 90.3 (13.8) | 90.4 (14.1) | 90.1 (13.0) | 0.752 |
| NIHSS at arrival, Median (IQR) | 2.0 (1.0, 3.0) | 2.0 (1.0, 3.0) | 2.0 (1.0, 4.0) | < 0.001 |
| Time after onset, mean (SD) | 25.7 (19.7) | 25.6 (20.0) | 25.9 (19.0) | 0.861 |
| Prior mRs, n (%) |  |  |  | 0.548 |
| 0 | 1063 (83.0) | 789 (83.7) | 274 (81.1) |  |
| 1 | 173 (13.5) | 122 (12.9) | 51 (15.1) |  |
| 2 | 45 ( 3.5) | 32 (3.4) | 13 (3.8) |  |
| Smoking status, n (%) |  |  |  | 0.754 |
| Never | 657 (52.6) | 478 (52) | 179 (54.2) |  |
| Previous smoker | 70 ( 5.6) | 53 (5.8) | 17 (5.2) |  |
| Current smoker | 523 (41.8) | 389 (42.3) | 134 (40.6) |  |
| Drinking, n (%) | 438 (35.0) | 315 (34.1) | 123 (37.3) | 0.304 |
| **Previous history** |  |  |  |  |
| Diabetes mellitus, n (%) | 387 (30.2) | 281 (29.8) | 106 (31.4) | 0.591 |
| Lipid disorder, n (%) | 32 ( 2.5) | 24 (2.5) | 8 (2.4) | 0.857 |
| Atrial fibrillation, n (%) | 8 ( 0.6) | 7 (0.7) | 1 (0.3) | 0.689 |
| TIA, n (%) | 24 ( 1.9) | 15 (1.6) | 9 (2.7) | 0.212 |
| Ischemic stroke, n (%) | 339 (26.5) | 241 (25.6) | 98 (29) | 0.219 |
| Carotid stenosis, n (%) | 7 ( 0.5) | 6 (0.6) | 1 (0.3) | 0.683 |
| **In-hospital treatment** |  |  |  |  |
| Antiplatelet therapy, n (%) |  |  |  | 0.786 |
| Single antiplatelet therapy | 493 (38.5) | 365 (38.7) | 128 (37.9) |  |
| Dual antiplatelet therapy | 788 (61.5) | 578 (61.3) | 210 (62.1) |  |
| Statin therapy, n (%) |  |  |  | 0.021 |
| Not used | 43 ( 3.4) | 30 (3.2) | 13 (3.8) |  |
| Normal dose | 521 (40.7) | 405 (42.9) | 116 (34.3) |  |
| Intensive dose | 717 (56.0) | 508 (53.9) | 209 (61.8) |  |
| Antihypertensive treatment | 833 (67.6) | 608 (67.2) | 225 (68.8) | 0.59 |
| Hypoglycemic treatment | 291 (22.8) | 203 (21.6) | 88 (26) | 0.095 |
| **Laboratory findings** |  |  |  |  |
| TG, mean (SD), mg/dL | 159.1 (111.5) | 157.5 (111.2) | 163.8 (112.3) | 0.376 |
| FBG, mean (SD), mg/dL | 121.9 (51.8) | 120.4 (51.1) | 126.1 (53.6) | 0.079 |
| LDL-C, mean (SD), mg/dL | 102.0 (32.8) | 100.8 (32.0) | 105.4 (34.6) | 0.027 |
| HDL-C, mean (SD), mg/dL | 39.9 (12.3) | 40.0 (13.0) | 39.7 (9.9) | 0.721 |
| TC, mean (SD), mg/dL | 164.9 (43.6) | 163.2 (42.6) | 169.4 (46.0) | 0.027 |
| HCY, mean (SD), mg/dL | 3.3 (3.2) | 3.3 (3.3) | 3.2 (3.1) | 0.577 |
| PLT, mean (SD), 10^3^/µL | 220.2 (66.7) | 220.2 (66.6) | 220.4 (66.9) | 0.965 |
| WBC, mean (SD), /µL | 7183.8 (2083.5) | 7086.9 (2068.6) | 7453.8 (2104.1) | 0.007 |
| Uric acid, mean (SD), mg/dL | 5.5 (1.6) | 5.6 (1.6) | 5.4 (1.6) | 0.175 |
| TyG, mean (SD) | 9.0 (0.7) | 8.9 (0.7) | 9.0 (0.7) | 0.047 |
| **Imaging features** |  |  |  |  |
| Multiple infarctions, n (%) | 447 (34.9) | 254 (26.9) | 193 (57.1) | < 0.001 |
| ICASB, Median (IQR) | 1.0 (0.0, 4.0) | 0.0 (0.0, 2.0) | 5.0 (3.0, 8.0) | < 0.001 |

**Table S4 Univariate logistic regression analysis of risk factors associated with sICAS.**

|  | **OR (95%CI)** | **P value** |
| --- | --- | --- |
| Age, year | 1.01 (1~1.02) | 0.067 |
| Male | 0.76 (0.58~0.99) | 0.039 |
| BMI | 1.01 (0.98~1.05) | 0.437 |
| NIHSS at arrival | 1.14 (1.05~1.23) | 0.001 |
| Systolic pressure, mmHg | 1 (1~1.01) | 0.21 |
| Diastolic pressure, mmHg | 1 (0.99~1.01) | 0.752 |
| Prior mRs (ref=0) |  |  |
| 1 | 1.2 (0.84~1.72) | 0.305 |
| 2 | 1.17 (0.61~2.26) | 0.641 |
| Smoking status (ref=never) |  |  |
| Previous smoker | 0.86 (0.48~1.52) | 0.596 |
| Current smoker | 0.92 (0.71~1.19) | 0.53 |
| Drinking | 1.15 (0.88~1.49) | 0.304 |
| Diabetes mellitus | 1.08 (0.82~1.41) | 0.591 |
| Lipid disorder | 0.93 (0.41~2.09) | 0.857 |
| Atrial fibrillation | 0.4 (0.05~3.24) | 0.388 |
| TIA | 1.69 (0.73~3.9) | 0.217 |
| Ischemic stroke | 1.19 (0.9~1.57) | 0.219 |
| Carotid stenosis | 0.46 (0.06~3.86) | 0.477 |
| TG, mmol/L | 1.04 (0.95~1.15) | 0.376 |
| FBG, mmol/L | 1.04 (1~1.08) | 0.08 |
| LDL-C, mmol/L | 1.18 (1.02~1.36) | 0.027 |
| HDL-C, mmol/L | 0.93 (0.62~1.39) | 0.721 |
| TC, mmol/L | 1.13 (1.01~1.26) | 0.027 |
| HCY, µmol/L | 1 (0.99~1) | 0.577 |
| PLT, 10^9^/L | 1 (1~1) | 0.965 |
| WBC, 10^9^/L | 1.09 (1.02~1.15) | 0.007 |
| Uric acid, µmol/L | 1 (1~1) | 0.175 |
| TyG as continuous | 1.19 (1~1.41) | 0.048 |
| TYG 2nd quantile (ref=1st) | 1.16 (0.81~1.66) | 0.412 |
| TYG 3rd quantile (ref=1st) | 1.02 (0.71~1.46) | 0.926 |
| TYG 4th quantile (ref=1st) | 1.45 (1.02~2.06) | 0.036 |
| ICASB, continuous | 1.66 (1.56~1.76) | <0.001 |
| ICASB categories |  |  |
| ICASB <4 |  |  |
| ICASB 4~5 | 7.79 (5.28~11.51) | <0.001 |
| ICASB >5 | 18.82 (13.22~26.81) | <0.001 |
| Multiple infarctions | 3.61 (2.79~4.68) | <0.001 |

*ORs and 95% CIs were expressed as per SI unit increase

**Table S5 Univariate liner regression analysis of risk factors associated with ICASB.**

|  | **β (95%CI)** | **P value** |
| --- | --- | --- |
| Age, year | 0.05 (0.04,0.07) | < 0.001 |
| Male | -0.45 (-0.86,-0.03) | 0.034 |
| BMI | -0.03 (-0.08,0.02) | 0.281 |
| NIHSS at arrival | 0.25 (0.13,0.37) | < 0.001 |
| Systolic pressure, mmHg | 0 (0,0.01) | 0.334 |
| Diastolic pressure, mmHg | -0.01 (-0.02,0) | 0.134 |
| Prior mRs (ref=0) |  |  |
| 1 | 0.41 (-0.15,0.96) | 0.151 |
| 2 | -0.21 (-1.24,0.81) | 0.684 |
| Smoking status (ref=never) |  |  |
| Previous smoker | -0.24 (-1.08,0.61) | 0.58 |
| Current smoker | -0.25 (-0.64,0.15) | 0.218 |
| Drinking | -0.16 (-0.56,0.24) | 0.443 |
| Diabetes mellitus | 0.56 (0.15,0.97) | 0.008 |
| Lipid disorder | -0.92 (-2.12,0.29) | 0.137 |
| Atrial fibrillation | -1.43 (-3.83,0.96) | 0.24 |
| TIA | 0.71 (-0.68,2.1) | 0.314 |
| Ischemic stroke | 0.43 (0.01,0.86) | 0.047 |
| Carotid stenosis | 0.17 (-2.39,2.72) | 0.899 |
| TG, mmol/L | -0.02 (-0.17,0.13) | 0.756 |
| FBG, mmol/L | 0.12 (0.06,0.19) | < 0.001 |
| LDL-C, mmol/L | 0.14 (-0.09,0.36) | 0.229 |
| HDL-C, mmol/L | 0.23 (-0.37,0.82) | 0.459 |
| TC, mmol/L | 0.05 (-0.12,0.22) | 0.568 |
| HCY, µmol/L | 0 (-0.01,0) | 0.223 |
| PLT, 10^9^/L | 0 (0,0) | 0.938 |
| WBC, 10^9^/L | 0.05 (-0.04,0.15) | 0.268 |
| Uric acid, µmol/L | 0 (0,0) | 0.079 |
| TyG as continuous | 0.29 (0.03,0.55) | 0.03 |
| TYG 2nd quantile (ref=1st) | 0.63 (0.1,1.16) | 0.02 |
| TYG 3rd quantile (ref=1st) | 0.52 (-0.01,1.05) | 0.055 |
| TYG 4th quantile (ref=1st) | 0.81 (0.27,1.34) | 0.003 |
| Multiple infractions | 1.33 (0.94,1.72) | < 0.001 |

*βs and 95% CIs were expressed as per SI unit increase

**Table S6 Subgroup analysis between TyG index and the degree of sICAS**

| **Variable** | **n.total** | **n.event (%)** | **Unadjusted OR (95% CI)** | **P value** | **Model I OR (95% CI)** | **P value** | **Model II OR (95% CI)** | **P value** |
| --- | --- | --- | --- | --- | --- | --- | --- | --- |
| TyG as continuous | 338 | 201 (59.5) | 1.34 (0.98~1.84) | 0.071 | 1.5 (1.07~2.09) | 0.018 | 1.48 (0.97~2.28) | 0.072 |
| TyG (categories) |  |  |  |  |  |  |  |  |
| TyG quartile 1 | 76 | 38 (50) | 1(Ref) |  | 1(Ref) |  | 1(Ref) |  |
| TyG quartile 2 | 85 | 50 (58.8) | 1.43 (0.77~2.66) | 0.262 | 1.5 (0.79~2.84) | 0.217 | 1.45 (0.7~3.01) | 0.313 |
| TyG quartile 3 | 77 | 47 (61) | 1.57 (0.82~2.98) | 0.17 | 1.74 (0.89~3.39) | 0.105 | 1.66 (0.77~3.59) | 0.194 |
| TyG quartile 4 | 100 | 66 (66) | 1.94 (1.05~3.58) | 0.033 | 2.35 (1.24~4.45) | 0.009 | 2.26 (1.02~5.04) | 0.046 |

Model I was adjusted for age and sex, and model II was adjusted for BMI, SBP, DBP, smoking status, drinking, previous stroke, and previous diabetes mellitus. Confounders were determined based on univariate analysis and previous literature reports.

**Table S7 Univariate Cox regression analysis of risk factors associated with ischemic stroke recurrence.**

|  | **HR (95%CI)** | **P value** |
| --- | --- | --- |
| Age, year | 0.9989 (0.9833,1.0148) | 0.895 |
| Female | 0.69 (0.48,1.01) | 0.055 |
| BMI | 1.0031 (0.9525,1.0563) | 0.907 |
| Systolic pressure, mmHg | 1.01 (1,1.02) | 0.007 |
| Diastolic pressure, mmHg | 1.0046 (0.9915,1.0179) | 0.493 |
| NIHSS at arrival | 1.11 (0.99,1.25) | 0.064 |
| Time after onset, hours | 0.9901 (0.9804,0.9999) | 0.049 |
| Prior mRs (ref=0) |  |  |
| 1 | 1.32 (0.81,2.13) | 0.263 |
| 2 | 0.47 (0.12,1.92) | 0.297 |
| Smoking status (ref=never) |  |  |
| Previous smoker | 0.56 (0.2,1.54) | 0.261 |
| Current smoker | 0.79 (0.54,1.17) | 0.239 |
| Drinking | 0.75 (0.5,1.12) | 0.157 |
| Diabetes mellitus | 2.2 (1.53,3.17) | < 0.001 |
| Lipid disorder | 2.7 (1.26,5.79) | 0.011 |
| Atrial fibrillation | 1.84 (0.26,13.19) | 0.543 |
| TIA | 1.38 (0.44,4.35) | 0.58 |
| Ischemic stroke | 1.13 (0.76,1.69) | 0.544 |
| Carotid stenosis | 1.54 (0.22,11.03) | 0.667 |
| Antiplatelet therapy |  | 0.423 |
| Single antiplatelet therapy | Reference |  |
| Dual antiplatelet therapy | 1.17 (0.8,1.71) |  |
| Statin therapy |  | 0.021 |
| Not used | Reference |  |
| Normal dose | 3.32 (0.46,24.17) |  |
| Intensive dose | 4.94 (0.69,35.52) |  |
| Antihypertensive treatment | 1.22 (0.81,1.84) | 0.331 |
| Hypoglycemic treatment | 0.78 (0.54,1.21) | 0.425 |
| TG, mmol/L | 0.92 (0.78,1.09) | 0.357 |
| FBG, mmol/L | 1.09 (1.04,1.15) | < 0.001 |
| LDL-C, mmol/L | 1.1 (0.9,1.35) | 0.367 |
| HDL-C, mmol/L | 1.06 (0.62,1.82) | 0.832 |
| TC, mmol/L | 1.03 (0.88,1.21) | 0.692 |
| HCY, µmol/L | 0.9972 (0.9884,1.006) | 0.526 |
| PLT, 10^9^/L | 1.001 (0.9984,1.0036) | 0.457 |
| WBC, 10^9^/L | 1.12 (1.03,1.21) | 0.007 |
| Uric acid, µmol/L | 0.9987 (0.9963,1.0012) | 0.305 |
| TyG, as continuous | 1.15 (0.9,1.47) | 0.254 |
| TYG, as quantiles |  |  |
| TyG quartile 1 | Reference |  |
|  | **HR (95%CI)** | **P value** |
| TyG quartile 2 | 1.73 (0.98,3.05) | 0.059 |
| TyG quartile 3 | 1.72 (0.98,3.04) | 0.061 |
| TyG quartile 4 | 1.84 (1.05,3.23) | 0.033 |
| ICASB, continuous | 1.07 (1.03,1.12) | 0.001 |
| ICASB categories |  |  |
| ICASB <4 |  |  |
| ICASB 4~5 | 1.67 (0.98,2.84) | 0.058 |
| ICASB >5 | 1.8 (1.18,2.76) | 0.007 |
| sICAS | 1.89 (1.31,2.74) | < 0.001 |
| Multiple infarctions | 1.69 (1.17,2.43) | 0.005 |

*HRs and 95% CIs were expressed as per SI unit increase

**Table S8 Sensitivity analysis after multiple imputation of variates with missing values.**

|  | **n.event (%)** | **Model III HR (95% CI)** | **P value** |
| --- | --- | --- | --- |
| TyG (continuous) | 117 (9.1) | 1.22 (0.96~1.55) | 0.096 |
| TyG (categories) |  |  |  |
| TyG quartile 1 | 19 (5.9) | 1(Ref) |  |
| TyG quartile 2 | 32 (10) | 1.65 (0.92~2.97) | 0.094 |
| TyG quartile 3 | 32 (10) | 1.88 (1.06~3.35) | 0.031 |
| TyG quartile 4 | 34 (10.6) | 2.11 (1.2~3.71) | 0.009 |
| ICASB (continuous) | 117 (9.1) | 1.07 (1.02~1.11) | 0.005 |
| ICASB (categories) |  |  |  |
| ICASB <4 | 70 (7.6) | 1(Ref) |  |
| ICASB 4~5 | 17 (12.4) | 1.81 (1.08~3.03) | 0.024 |
| ICASB >5 | 30 (13.5) | 1.66 (1.07~2.58) | 0.023 |
| sICAS |  |  |  |
| Without sICAS | 70 (7.4) | 1(Ref) |  |
| With sICAS | 47 (13.9) | 1.72 (1.18~2.5) | 0.005 |

Model was adjusted for age, sex, BMI, SBP, smoking status, time at onset, NIHSS score at arrival, WBC, previous stroke, previous diabetes mellitus, antiplatelet therapy and intensive statin therapy.

**Table S9 Interaction effect on stroke recurrence between TyG and sICAS**

| **Variable** | **n.event (%)** | **crude HR (95% CI)** | **crude P value** | **adj.HR (95% CI)** | **adj.P value** | **P for interaction** |
| --- | --- | --- | --- | --- | --- | --- |
| TyG as continuous |  |  |  |  |  |  |
| Without sICAS | 70 (7.4) | 1.1 (0.81~1.51) | 0.537 | 1.05 (0.72~1.52) | 0.808 | 0.63 |
| With sICAS | 47 (13.9) | 1.31 (0.89~1.93) | 0.173 | 1.29 (0.8~2.08) | 0.302 |  |
|  |  |  |  |  |  |  |
| TyG as quartiles |  |  |  |  |  |  |
| Without sICAS |  |  |  |  |  | 0.964 |
| TyG quartile 1 | 14 (5.7) | 1(Ref) |  | 1(Ref) |  |  |
| TyG quartile 2 | 16 (6.8) | 1.2 (0.59~2.46) | 0.617 | 1.13 (0.52~2.45) | 0.756 |  |
| TyG quartile 3 | 21 (8.6) | 1.53 (0.78~3.02) | 0.215 | 1.54 (0.74~3.21) | 0.253 |  |
| TyG quartile 4 | 19 (8.6) | 1.55 (0.78~3.09) | 0.214 | 1.42 (0.64~3.14) | 0.382 |  |
| With sICAS |  |  |  |  |  |  |
| TyG quartile 1 | 7 (9.2) | 1(Ref) |  | 1(Ref) |  |  |
| TyG quartile 2 | 11 (12.9) | 1.48 (0.57~3.82) | 0.417 | 1.5 (0.53~4.24) | 0.449 |  |
| TyG quartile 3 | 12 (15.6) | 1.81 (0.71~4.59) | 0.213 | 1.7 (0.61~4.77) | 0.312 |  |
| TyG quartile 4 | 17 (17) | 1.94 (0.81~4.69) | 0.139 | 1.98 (0.72~5.49) | 0.187 |  |
|  |  |  |  |  |  |  |
| TyG as dichotomized |  |  |  |  |  |  |
| Without sICAS |  |  |  |  |  | 0.043 |
| TYG<8.4 | 14 (6.3) | 1(Ref) |  | 1(Ref) |  |  |
| TYG2≥8.4 | 56 (7.8) | 1.24 (0.69~2.23) | 0.468 | 1.23 (0.66~2.3) | 0.519 |  |
| With sICAS |  |  |  |  |  |  |
| TYG<8.4 | 5 (7.2) | 1(Ref) |  | 1(Ref) |  |  |
| TYG2≥8.4 | 42 (15.6) | 2.29 (1.21~5.79) | 0.03 | 3.09 (1.17~10.92) | 0.027 |  |

Model was adjusted for age, sex, BMI, SBP, smoking status, time at onset, NIHSS score at arrival, WBC, previous stroke, previous diabetes mellitus, antiplatelet therapy, intensive statin therapy, hypoglycemic treatment, and antihypertensive treatment. Cut-off value of TyG was determined by X-tile.

**Table S10 The value of TyG improved the risk stratification of stroke recurrence according to continuous-NRI and IDI**

| **Comparison** | **Continuous NRI** |  |  | **IDI** |  |
| --- | --- | --- | --- | --- | --- |
|  | **Est. (95% CI)** | **p value** |  | **Est, (95% CI)** | **p value** |
| Model I | Ref |  |  | Ref |  |
| Model II | 0.188 (0.066-0.268) | <0.001 |  | 0.01 (0.003-0.03) | <0.001 |

Model I included conventional risk factors (age, gender, BMI, SBP, smoking status, time from onset, NIHSS score, WBC, previous stroke, diabetes, sICAS, antiplatelet treatment, intensive statin treatment, antihypertensive treatment and hypoglycemic treatment) according to the univariate regression results and literature reports. Model II included TyG index further.

**2. Additional file 1 figure list:**


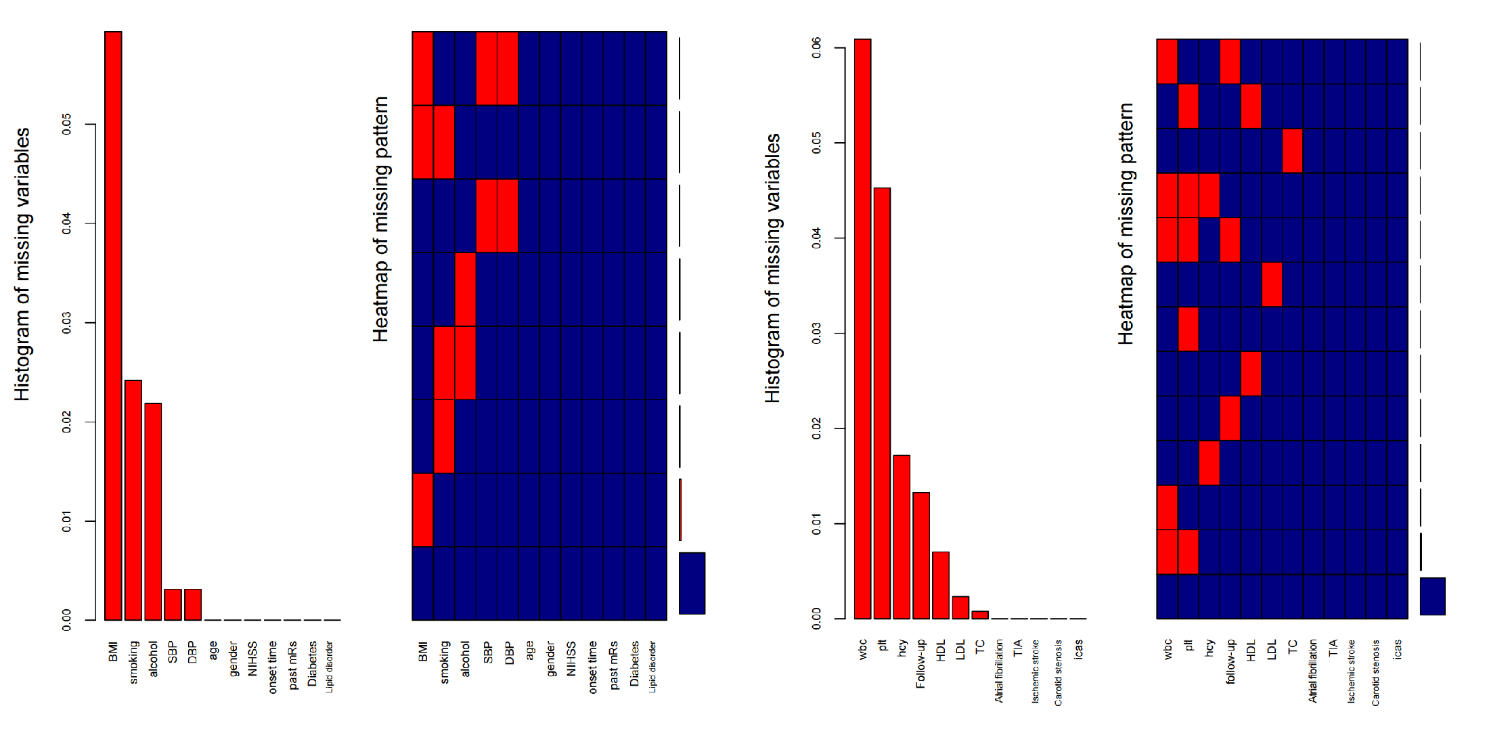


**Figure S1 Pattern of missing data.**

Variables with missing data were displayed in histogram and heat plot (WBC, BMI, PLT, smoking, drinking, HCY, follow-up, HDL, SBP, DBP, LDL, TC, age, NIHSS, gender, onset time, diabetes, lipid disorder, past mRs, ICAS, atrial fibrillation, previous ischemic stroke, TIA, carotid stenosis). The data were missing at random.


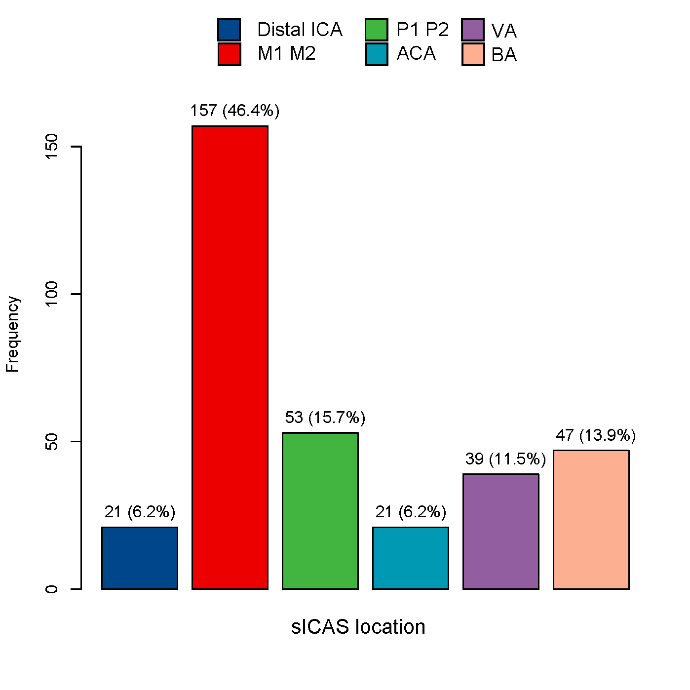


**Figure S2 Distribution of arteries responsible for sICAS.**

M1 and M2 segments were the most represented responsible arteries among patients with sICAS.


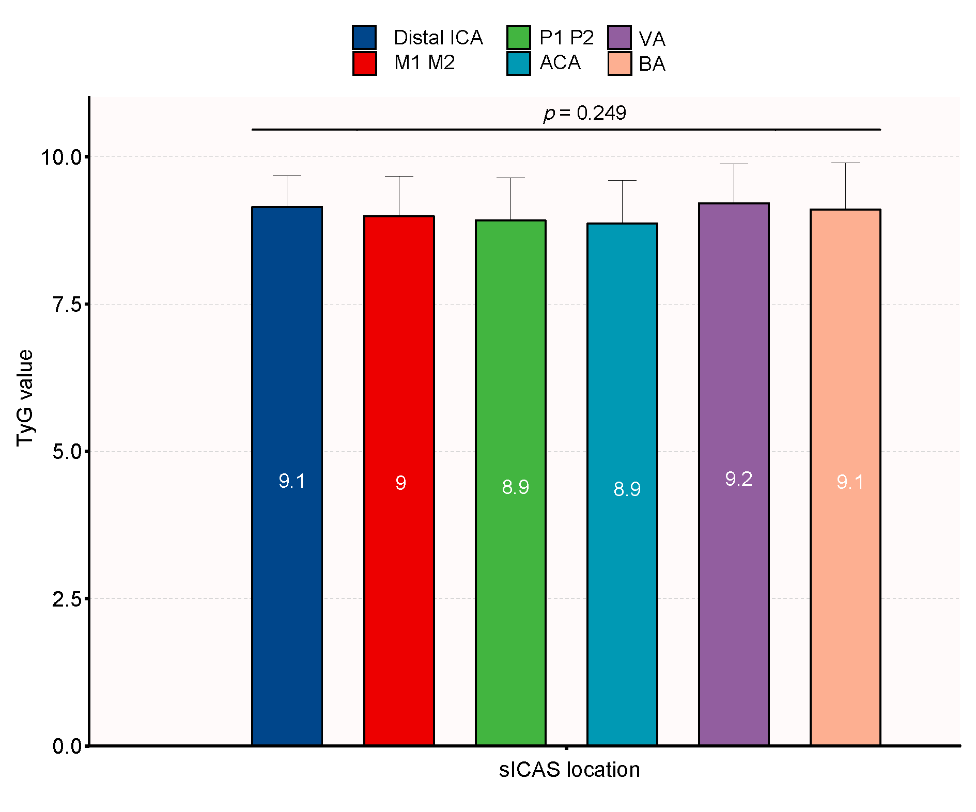


**Figure S3 Association between responsible sICAS arteries and TyG.**

No significant difference in TyG levels was found among the different types of responsible artery groups.


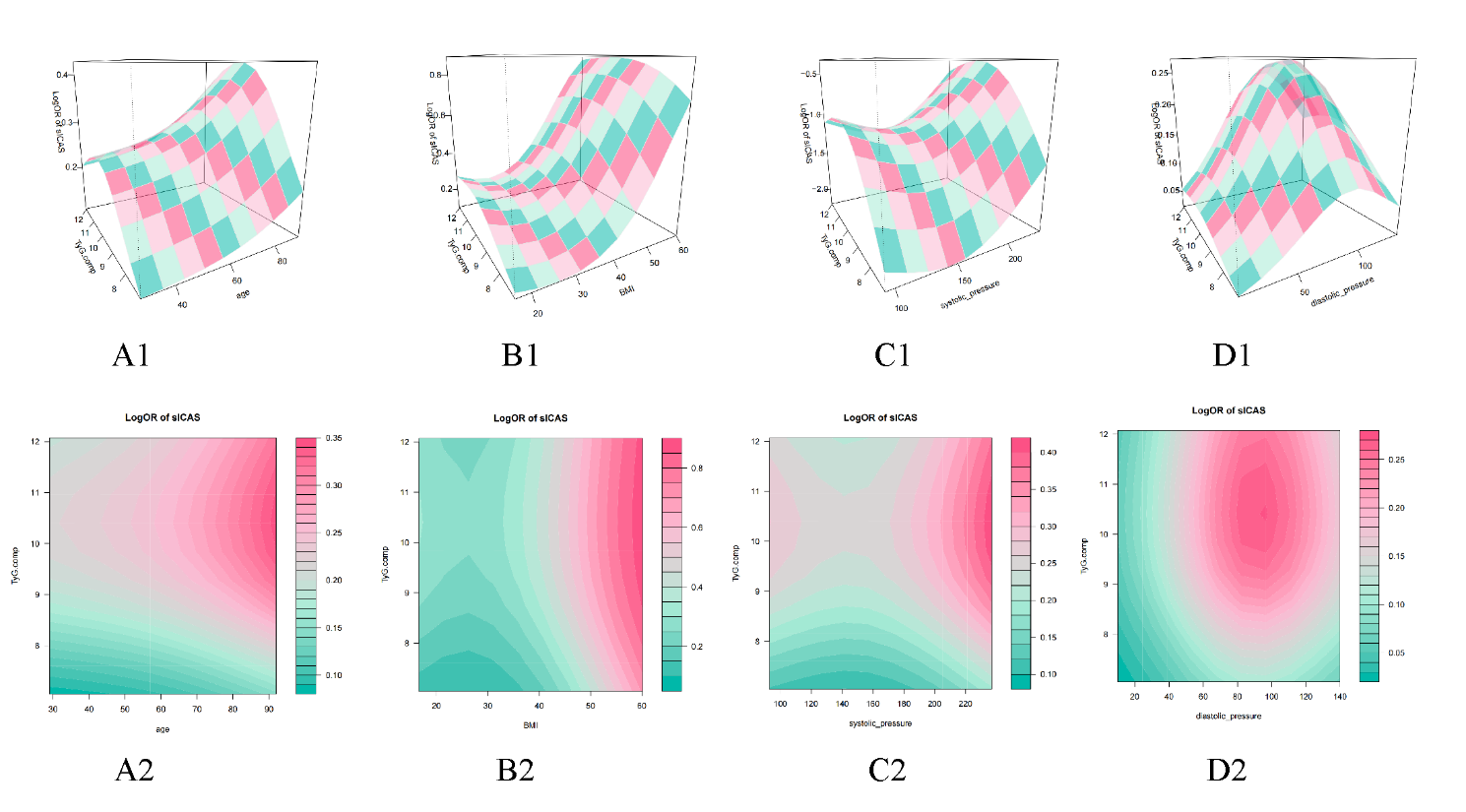


**Figure S4 Contour plots reflecting the effect of age, BMI, and blood pressure on the relationship between TyG and sICAS.**

A1&A2: the effect of age; B1&B2: the effect of BMI; C1&C2: the effect of SBP; D1&D2: the effect of DBP.


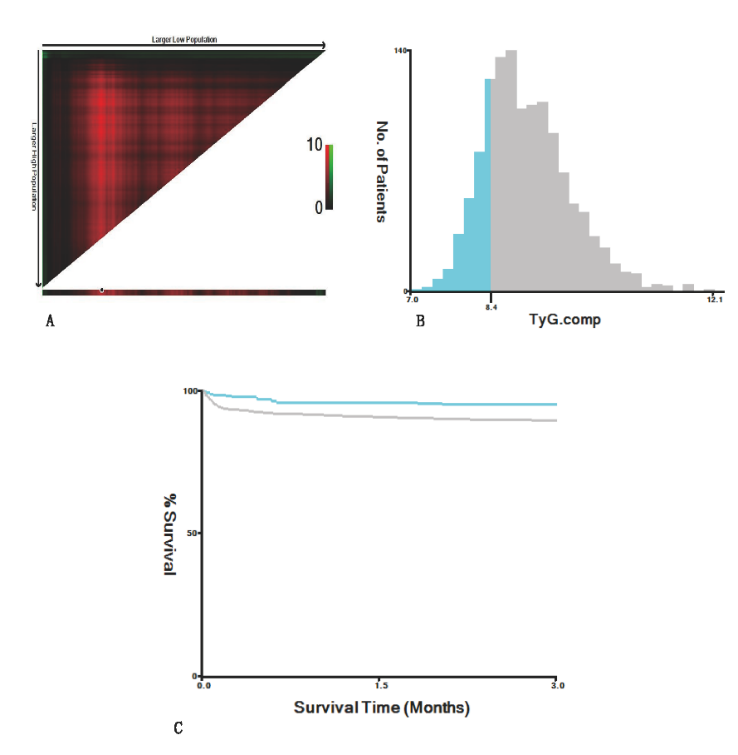


**Figure S5 X tile analyses of TyG optimal cut-off value.**

A. X-tile plots for minor ischemic stroke patients with TyG. Black circle refers to the optimal threshold value of TyG.

B. Histogram of TyG distribution and optimal threshold value of TyG.

C. Survival curves of patients with low and high TyG stratified by the threshold.


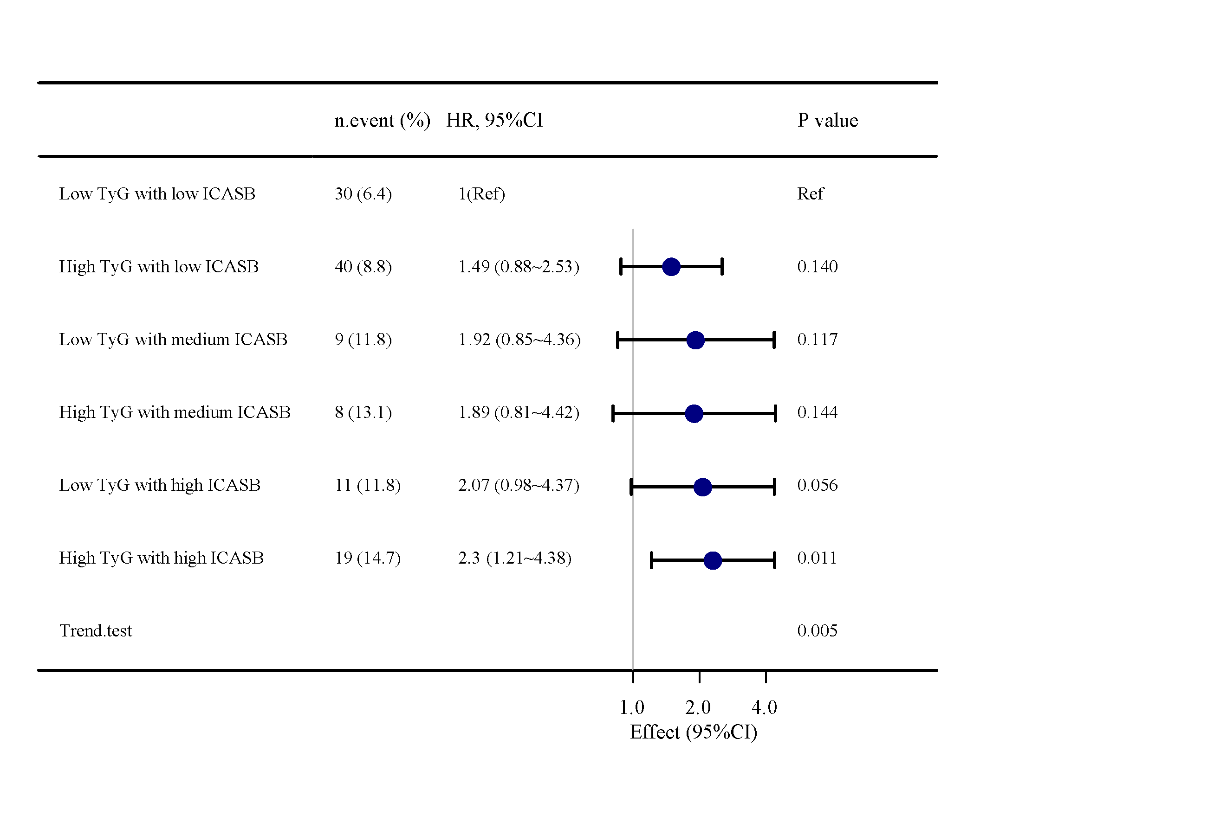


**Figure S6 Combined effect of TyG and burden of ICAS on ischemic stroke recurrence.**

Low ICASB: <4 scores; Medium ICASB: 4-5 scores; High ICASB: >5 scores. Patients with high TyG and high ICASB had the highest risk of recurrence.


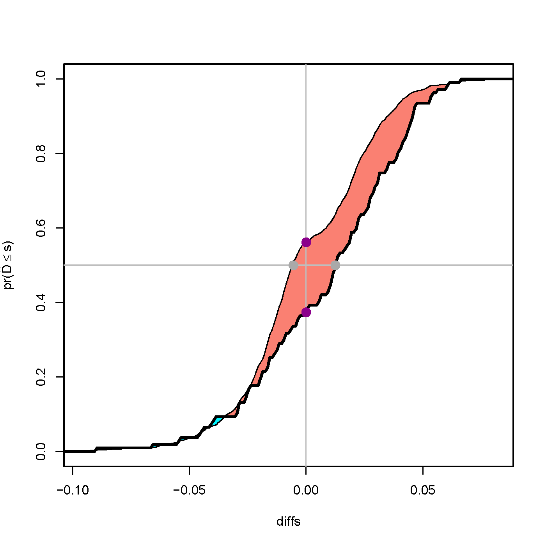


**Figure S7 IDI plot.**

The area of the red part minus the area of the blue part is IDI. The distance between two purple dots represents continuous NRI, and the distance between two gray dots represents median improvement in risk score.
